# Patterns of use of wild food plants by Brazilian local communities: systematic review and meta-analysis

**DOI:** 10.1186/s13002-023-00619-y

**Published:** 2023-10-25

**Authors:** Lailson César Andrade Gomes, Patrícia Muniz de Medeiros, Ana Paula do Nascimento Prata

**Affiliations:** Campus of Engineering and Agricultural Sciences of the Federal University of Alagoas, Rio Largo, Brazil

**Keywords:** Ethnobotanical hypotheses, Wild food plants, Food resource management

## Abstract

**Background:**

This systematic review and meta-analysis sought to investigate the patterns of use of native wild food plants of Brazil (native and non-cultivated).

**Methods:**

We searched ethnobiological works with food plants in Web of Science, Scielo, Scopus and PubMed using different sets of keywords. Initially, the studies were evaluated based on inclusion criteria (systematic data collection instruments, such as interviews; specification of methods for data collection; and the presence of a species list). The methodological quality of each study was evaluated to define the risk of bias. A total of 20 articles met all criteria and were included in the review.

**Results:**

The results showed that there was a predominance of consumption of fruits, followed by leaves and seeds, which together represented 85.8% of the total parts. As for the meta-analysis, there was a predominance of use of plant parts classified as reproductive, non-persistent, non-destructive and parts of woody plants. There was no interference from the type of ecosystem (seasonally dry *x* moist). The results did not support the seasonality hypothesis.

**Conclusions:**

The concentration of studies in the Northeast, Southeast and South regions of Brazil and in the Atlantic Forest and Caatinga biomes points to the need for a greater effort in terms of quantitative ethnobotanical research in other regions and biomes. The predominance of fruits and plant parts classified as reproductive, non-persistent and non-destructive points to the high potential for implementation of sustainable management strategies aimed at these plants in the country.

**Supplementary Information:**

The online version contains supplementary material available at 10.1186/s13002-023-00619-y.

## Introduction

Human populations make use of plant resources in their surroundings taking into account their own particularities and needs. However, despite the idiosyncrasies, some behaviors are recurrent in different socio-ecological systems. This may be due, for example, to cultural, environmental or historical similarities that lead people from different places to use plants in a similar way [1]. Thus, not only the study of general trends in how people get hold of plant resources, but also of use patterns can shed light on the factors that explain the differences. For example, some studies have identified the important role of ethnicity or the ecosystem in plant use [2–4].

Ethnobiology provides plenty of information on people-plant relationships that can contribute to unravel patterns. Identifying these patterns, in turn, is relevant from a theoretical point of view, assisting in the understanding of certain aspects of the relationship between people and plants, but also from a practical point of view when applied to decision-making, especially in the management of plant resources. In this sense, understanding the patterns can be crucial to evaluate the ecological impact of plant resource management.

Over the past decade, the number of ethnobiological studies aiming to identify use patterns on scales larger than the local scale has grown significantly. These studies have been directed mainly to medicinal plants [3, 5], multiple-use species [4, 6, 7] or a single useful species [2, 8]. In the case of food plants, there are studies on use patterns at small scales (municipality, district, etc.) [9], but efforts on larger scales are still necessary. The present work aims to fill this gap by identifying patterns of use of wild food plants among local Brazilian populations. The species may have a significant role in the food and nutritional security of these populations, in addition to contributing to income generation for local farmers and extractivists.

The understanding of the main forms of appropriation of wild food plants at a large scale may indicate which ecological processes could be more compromised in cases of overexploitation or even allow more general conclusions about the potential for sustainable use of these resources. For example, the predominant use of reproductive parts (flowers, fruits and seeds) can especially affect the recruitment of new individuals [10, 11], so that the damage to the plant population brought about by an eventual overexploitation may not be necessarily observed in the short term [12]. Still, from the point of view of pressure of use, the predominance of collection of parts whose extraction has the potential to destroy the individual, such as roots or stems, is expected to cause a rapid deterioration of populations. Thus, the identification of regional or national trends can assist in the proposal of more comprehensive conservation strategies without disregarding the need for policies aimed at local specificities.

Another trend that can be explored in the study of use patterns relates to the strategies adopted by human populations to select plant resources. For example, the seasonality hypothesis [13], addressed within the scope of the availability hypothesis in some studies [14], indicates that, in seasonal environments, people will channel their attention to resources that are more likely to persist throughout the year, enhancing the security in resource acquisition. For example, in arid and semiarid environments, some plant parts (e.g., leaves) are more likely to be lost during the dry season. In addition, herbs would be less likely to persist during the dry period that woody species (shrubs and trees). Therefore, people would focus their attention on persistent plant parts (e.g., roots) and perennial species.

Studies that addressed this hypothesis at different scales are focused on medicinal plants [3, 15].

In the present study, we conducted a systematic review on a national scale (Brazil) to investigate use patterns associated with the habit and parts of wild food plants.

Therefore, this work sought to answer the following questions:

1. How are works with an ethnobotanical approach distributed in Brazil?
2. What parts of wild food plants occurring in Brazil are predominantly used and documented in the literature?
3. Is the consumption of wild food plants by local Brazilian populations predominantly focused on reproductive or non-reproductive parts?
4. Is the consumption of wild food plants by local Brazilian populations predominantly focused on destructive or non-destructive parts?
5. Is the consumption of wild food plants by local Brazilian populations predominantly focused on persistent or non-persistent parts?
6. Is the consumption of wild food plants by local Brazilian populations predominantly focused on woody or non-woody plants?
7. Is the use of persistent parts and woody plants more predominant in seasonal environments or in non-seasonal environments?

Our methodological approach considered only native species; food plants that are considered wild plants because they are naturalized were excluded from this review.

## Methodology

Studies investigating broad-scale use patterns rely on primary [2, 6, 8] or secondary [3, 5, 7] data. The use of primary data is advantageous in terms of methodological quality, since the same research design is applied to the different sites. However, the enormous sampling effort and the logistics involved cause many investigations to lack sampling robustness at the community level. The use of secondary data can circumvent this problem, but there is the barrier of lack of methodological uniformity (which can eventually incorporate bias). Further, the use of secondary data depends on the amount of studies developed at the target site. Thus, the present review adopted exclusion criteria based on the risk of bias of the studies in order to reduce the problems typical of investigations relying on secondary data.

### Bibliographic search

Scientific articles with an ethnobotanical approach that presented a list of food plants occurring in Brazil were sought. To this end, four databases were consulted: Web of Science, Scielo, Scopus and PubMed. Search queries were performed using the following pre-established keywords in English and Portuguese: (1) “Unconventional Food Plants” AND Brazil; (2) “Wild Food Plants” AND Brazil; (3) “Wild Edible Plants” AND Brazil; (4) “Useful Plants” AND Ethnobotany AND Brazil; (5) “Plantas Comestíveis” AND Brasil; (6) “Plantas Alimentícias Não Convencionais” AND Brasil; (7) “Plantas Alimentícias Silvestres” AND Brasil; (8) “Plantas Úteis” AND Etnobotânica AND Brasil. The searches were carried out by title, abstract and keywords of the articles in the period from March 11, 2022, to March 15, 2022.

### Screening and exclusion criteria

In a first screening, duplicates, that is, articles found more than once in different databases (Web of Science, Scielo, Scopus and PubMed), were excluded; they were entered only once in the database. Then, in a second screening, the abstract of each article was read and articles without an ethnobotanical approach were excluded. Review articles were excluded, but their references were used to search for articles with primary data. Studies that were not published in Portuguese or English were also excluded. Works with more general approaches (multiple uses) were selected for later extraction of data pertaining to food plants.

A third screening was performed. The articles included during the previous stages were read in full length. Those that did not present a list of species and those that did not present the scientific names of the species were excluded. In cases of two or more studies conducted in the same community or using the same database, only the one that contained more complete and detailed information was included. Also, only studies that used systematic data collection instruments, such as interviews, were considered. Studies that did not have information about the data collection methodology were excluded.

### Selection of studies based on risk of bias

Articles selected after application of inclusion/exclusion criteria and screening steps were classified as presenting low, moderate and high risk of bias (Additional file 1), according to criteria to establish the risk of bias in ethnobotanical studies of medicinal plants based on sample quality [16]. It is important to note that the classification of risk of bias is not intended to judge the merit and quality of the ethnobiological studies, since the samples also depend on the theoretical and epistemological orientation of the researchers. It is possible, for example, that a study with a qualitative nature whose theoretical sample is consistent and adequate to its objectives is classified here as having a high risk of bias simply because the data do not allow the identification of local trends that are necessary for the composition of a general framework.

Articles presenting moderate and low risk underwent another classification in order to evaluate a possible increase in the risk of bias based on the following information: complete or incomplete identification of plant material; presentation of a complete or partial list of species; and presence of restrictions in the studied habit or taxonomic groups, for example, studies conducted only with herbs or forest species or studies conducted only with one family [3].

In addition, in order to perform the meta-analysis, data on species to which information on the part used was not provided and articles containing less than five species were excluded. Finally, articles classified as presenting moderate and low risk were included in the analysis and the others were removed.

### Data treatment

Data on food species and places where the studies were conducted were extracted from each article. The following information was collected: bibliographic reference, biome, region, state, scientific name, family, popular name, part used and form of use.

In addition, information on all species occurring in Brazil was extracted using the flora package in R [17]. The information included: scientific name, family, life form, habitat, type of vegetation and establishment (origin) according to the listing of Flora do Brasil [18]. The correct spelling and accepted names of the species were checked also using this database. When a species was not mentioned in the listing of Flora do Brasil, the database World Flora Online was consulted [19].

Finally, only the list of accepted native Angiosperm species was extracted from the listings of Flora do Brasil [18] and World Flora Online [19]. Species considered naturalized, exotic, cultivated and those without the source information were excluded.

The plant parts used were classified into persistent (stem and root) or non-persistent (leaves, flowers, fruits, pseudofruits and seeds) [3]. In the second analysis, the parts were classified into reproductive (flowers, fruits, pseudofruits and seeds) or non-reproductive (leaf, stem and root). In the third analysis, the parts were classified into destructive (root, stem, whole plant, underground organ and aerial part) or non-destructive (leaves, flowers, fruits, pseudofruits and seeds). Finally, the species were classified as woody and non-woody; palm trees were excluded because they have a tree-like habit and are monocotyledonous.

### Data analysis

To understand the distribution of studies with wild food plants in Brazil, the locations where the works were conducted were extracted from the geographical coordinates of the municipalities mentioned in the studies and a distribution map of those with low or moderate risk of bias was prepared.

The main parts used for food purposes cited in the studies were summarized through descriptive statistics. Meta-analytical tools were employed in order to seek regularities among the studies in the nature of these parts (persistent *x* non-persistent, reproductive *x* non-reproductive, destructive *x* non-destructive) and the habit of plants (woody *x* non-woody).

Initially, we recorded the number of species in each study whose used parts were reproductive and non-reproductive parts, with the possibility of repetition in case a single species had at least one reproductive and one non-reproductive part. Then, the effect size was calculated for each study, using reproductive parts as reference, based on the ‘scalc’ function of the ‘metafor’ package in R [20]. PLO was the measurement option used (logit-transformed proportion). Then, a random effects model was performed with the ‘rma’ function of the ‘metafor’ package in R [20]. The same procedure was done for the other cases (destructive *x* non-destructive parts, persistent *x* non-persistent parts and woody *x* non-woody species).

To investigate whether there was a higher proportion of use of woody species and persistent parts in seasonally dry environments, as suggested by the seasonality hypothesis, the studies were classified according to the ecosystem where they were carried out, either seasonally dry environments or moist environments. Seasonally dry environments included areas located in the domain of Caatinga, while moist environments included the Atlantic Forest (moist forest) and Pantanal (seasonally flooded forest). We chose to evaluate the hypothesis by comparing seasonally dry and moist environments because we understand that, in the former, there is a greater shortage of non-woody plants and non-persistent parts during the dry season.

A mixed effects model was run using the ‘rma’ function, with the type of ecosystem (seasonally dry *x* moist) as a moderating factor. The analysis was made for the two variables associated with the hypothesis of seasonality (habit and persistence) and, in an exploratory way, also for the other variables.

A forest graph to display the results was made using the ‘forest.rma’ function of the ‘metfor’ package [20].

## Results

**Fig. 1:**
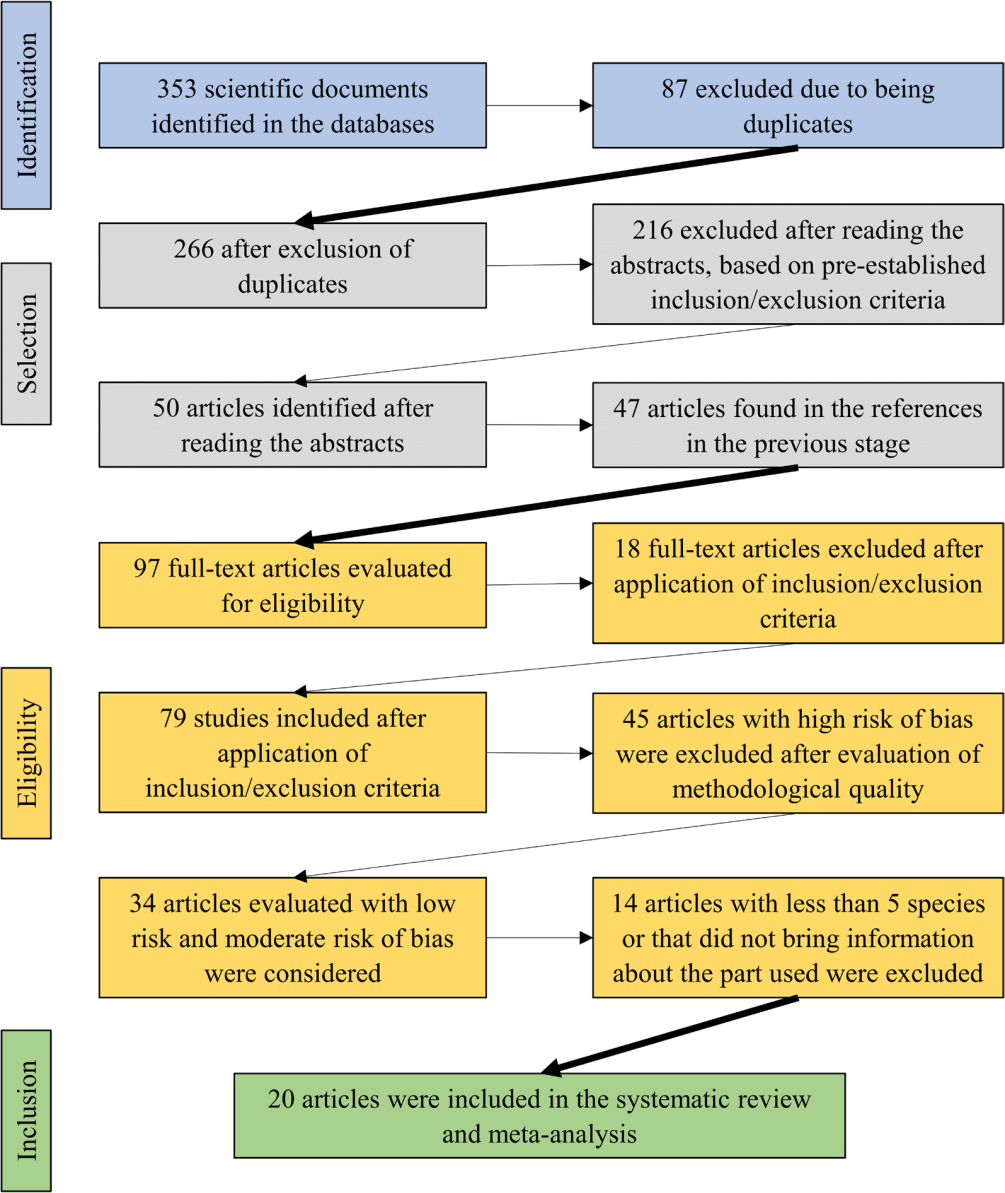
Process of selection of articles included in the systematic review and meta-analysis

Figure 1 shows the processes of identification, selection, eligibility and inclusion of articles in the systematic review and meta-analysis.

Seventy-nine articles met the inclusion criteria of this review (Additional file 2). However, 45 of them were considered to present a high risk of bias, 17 a moderate risk, and 19 a low risk, leaving 34 articles. Then, articles that contained less than 5 species and those that did not bring the information about the parts used were excluded, leaving 20. The articles included in this review are shown in Table 1.

**Table 1:** Listing and general aspects of studies with an ethnobotanical approach including wild food plants in Brazil

Article	State	Region	Ecosystem	Community type	Area
Baptista et al. [21]	Rio Grande do Sul	S	AF	Artisanal fishermen	U
Borges and Peixoto [22]	Rio de Janeiro	SE	AF	Caiçaras^1^	R
Bortolotto et al. [23]	Mato Grosso do Sul	MW	PAN	Rural	R
Brito and Senna-Valle [24]	Rio de Janeiro	SE	AF	Caiçaras	S/i
Campos et al. [25]	Ceará	NO	CA	Extractivists	R
Chaves et al. [26]	Piauí	NO	CA	Rural	R
Christo et al. [27]	Rio de Janeiro	SE	AF	Rural	R
Conde et al. [28]	Minas Gerais	SE	AF	Quilombola^2^	R
Crepaldi and Peixoto [29]	Espírito Santo	SE	AF	Quilombola	R
Fonseca-Kruel and Peixoto [30]	Rio de Janeiro	SE	AF	Artisanal fishermen	U
Leal et al. [31]	Santa Catarina	S	AF	Rural	U
Lobo et al. [32]	Pernambuco	NO	AF	Gypsies	N/i
Lopes and Lobão [33]	Espírito Santo	SE	AF	Artisanal fishermen	R
Medeiros et al. [34]	Alagoas	NO	AF	Farmers	R
Moura et al. [35]	Sergipe	NO	AF	Artisanal fishermen	R
Nascimento et al. [36]	Pernambuco	NO	CA	Rural	R
Nascimento et al. [37]	Pernambuco	NO	CA	Rural	R
Nunes et al. [38]	Paraíba	NO	CA	Rural	R
Roque and Loiola [39]	Rio Grande do Norte	NO	CA	Rural	R
Tuler et al. [40]	Minas Gerais	SE	AF	Farmers	R

Region: S—South, SE—Southeast, MW—Midwest, NO—Northeast, N—North. Ecosystem: AF—Atlantic Forest, PAN—Pantanal, CA—Caatinga. Area: U—Urban, R—Rural. N/i—No information. ^1^Traditional inhabitants of the coast of Southeastern Brazil; ^2^Descendants of Afro-Brazilian runaway slaves living in hideouts up-country called Quilombos

The list of species found in the articles that were included in the meta-analysis, as well as information about the parts used, can be found in Table 2.

**Table 2:** List of species found in articles that were included in the meta-analysis

Family	Scientific name	Used part	Woody *x* Non-woody
Alismataceae	*Echinodorus grandiflorus* (Cham. & Schltr.) Micheli	Leaf	Non-Woody
Anacardiaceae	*Spondias mombin* L.	Fruit	Woody
	*Anacardium occidentale* L.	Fruit	Woody
	*Schinus terebinthifolia* Raddi	Leaf, Fruit, Pseudofruit, Seed	Woody
	*Spondias tuberosa* Arruda	Root	Woody
	*Spondias macrocarpa* Engl.	Fruit	Woody
Annonaceae	*Annona cornifolia* A.St.-Hil.	Fruit	Woody
	*Annona nutans* (R.E.Fr.) R.E.Fr.	Fruit	Woody
	*Annona mucosa* Jacq.	Fruit	Woody
	*Annona coriacea* Mart.	Fruit	Woody
	*Duguetia furfuracea* (A.St.-Hil.) Saff.	Fruit	Woody
	*Annona glabra* L.	Fruit	Woody
	*Annona dolabripetala* Raddi	Fruit	Woody
Apiaceae	*Eryngium foetidum* L.	Leaf	Non-Woody
Apocynaceae	*Hancornia speciosa* Gomes	Fruit	Woody
	*Allamanda cathartica* L.	Leaf	Woody
	*Mandevilla tenuifolia* (J.C.Mikan) Woodson	Stem, Root	Woody
Aquifoliaceae	*Ilex paraguariensis* A.St.-Hil.	Leaf	Woody
Asteraceae	*Hypochaeris chillensis* (Kunth) Britton	Whole Plant	Non-Woody
	*Mikania glomerata* Spreng.	Fruit	Woody
	*Vernonanthura polyanthes* (Sprengel) Vega & Dematteis	Leaf	Woody
	*Sonchus oleraceus* L.	Seed, Leaf	Non-Woody
	*Erechtites valerianifolius* (Wolf) DC.	Leaf	Non-Woody
Basellaceae	*Anredera cordifolia* (Ten.) Steenis	Leaf	Woody
Bignoniaceae	*Tynanthus cognatus* (Cham.) Miers	Stem	Woody
Bixaceae	*Bixa orellana* L.	Leaf, Seed	Woody
Boraginaceae	*Varronia curassavica* Jacq.	Fruit	Woody
	*Varronia polycephala* Lam.	Fruit	Woody
	*Varronia globosa* Jacq.	Fruit	Woody
Bromeliaceae	*Bromelia antiacantha* Bertol.	Fruit	Non-Woody
	*Ananas comosus* (L.) Merril	Fruit	Non-Woody
	*Ananas ananassoides* (Baker) L.B.Sm.	Fruit	Non-Woody
	*Bromelia laciniosa* Mart. ex Schult. & Schult.f	Leaf	Non-Woody
	*Neoregelia cruenta* (R.Graham) L.B.Sm.	Fruit	Non-Woody
	*Aechmea comata* (Gaudich.) Baker	Fruit, Leaf, Flower	Non-Woody
	*Ananas bracteatus* (Lindl.) Schult. & Schult.f.	Fruit	Non-Woody
	*Dyckia spectabilis* (Mart. ex Schult. & Schult.f.) Baker	Leaf, Pseudofruit, Fruit	Non-Woody
Cactaceae	*Cereus jamacaru* DC.	Fruit	Woody
	*Xiquexique gounellei* (F.A.C.Weber) Lavor & Calvente	Fruit	Woody
	*Cereus bicolor* Rizzini & A.Mattos	Fruit	Woody
	*Melocactus zehntneri* (Britton & Rose) Luetzelb.	Stem	Woody
	*Pereskia aculeata* Mill	Leaf	Woody
	*Brasiliopuntia brasiliensis* (Willd.) A.Berger	Fruit	Woody
	*Pilosocereus arrabidae* (Lem.) Byles & Rowley	Fruit	Woody
	*Rhipsalis teres* (Vell.) Steud.	Fruit, Stem	Woody
	*Pilosocereus pachycladus* F.Ritter	Stem, Fruit	Woody
	*Tacinga inamoena* (K.Schum.) N.P.Taylor & Stuppy	Fruit	Woody
Capparaceae	*Crateva tapia* L.	Stem, Fruit	Woody
	*Cynophalla flexuosa* (L.) J.Presl	Fruit	Woody
	*Neocalyptrocalyx longifolium* (Mart.) Cornejo & Iltis	Fruit	Woody
Caricaceae	*Jacaratia spinosa* (Aubl.) A.DC	Stem	Woody
Caryocaraceae	*Caryocar brasiliense* Cambess.	Fruit	Woody
	*Caryocar coriaceum* Wittm.	Fruit, Seed	Woody
Celastraceae	*Salacia elliptica* (Mart.) G. Don	Fruit	Woody
	*Monteverdia rigida* (Mart.) Biral	Fruit	Woody
Chrysobalanaceae	*Couepia uiti* (Mart. & Zucc.) Benth. ex Hook.f	Fruit	Woody
	*Chrysobalanus icaco* L.	Fruit, Leaf	Woody
	*Hirtella corymbosa* Cham. & Schltdl.	Fruit	Woody
	*Couepia rufa* Ducke	Fruit	Woody
Clusiaceae	*Garcinia gardneriana* (Planch. & Triana) Zappi	Fruit	Woody
	*Garcinia brasiliensis* Mart.	Fruit	Woody
Combretaceae	*Terminalia corrugata* (Ducke) Gere & Boatwr.	Fruit	Woody
Cucurbitaceae	*Cucumis anguria* L.	Fruit	Woody
Cyperaceae	*Cyperus pedunculatus* (R.Br.) J.Kern	Root	Non-Woody
Dioscoreaceae	*Dioscorea trifida* L.f	Stem	Woody
	*Dioscorea coronata* Hauman	Root, Stem	Woody
Ebenaceae	*Diospyros lasiocalyx* (Mart.) B.Walln.	Fruit	Woody
	*Diospyros inconstans* Jacq.	Fruit	Woody
Ericaceae	*Gaylussacia brasiliensis* (Spreng.) Meisn.	Fruit	Woody
Euphorbiaceae	*Manihot esculenta* Crantz	Root, Leaf	Woody
	*Microstachys corniculata* (Vahl) Griseb.	Fruit	Woody
	*Manihot dichotoma* Ule	Stem, Root	Woody
	*Manihot glaziovii* Müll.Arg.	Root	Woody
	*Cnidoscolus quercifolius* Pohl	Fruit, Seed	Woody
	*Cnidoscolus urens* (L.) Arthur	Seed	Woody
Fabaceae	*Inga sessilis* (Vell.) Mart.	Fruit	Woody
	*Hymenaea stigonocarpa* Mart. ex Hayne	Seed	Woody
	*Inga vera* Willd	Fruit	Woody
	*Senna occidentalis* (L.) Link	Seed	Woody
	*Hymenaea courbaril* L.	Seed	Woody
	*Inga striata* Benth.	Fruit	Woody
	*Macropsychanthus grandiflorus* (Mart. ex Benth.) L.P.Queiroz & Snak	Seed	Woody
	*Hymenaea martiana* Hayne	Fruit	Woody
	*Swartzia flaemingii* Raddi	Fruit	Woody
	*Senna alata* (L.) Roxb.	Seed	Woody
	*Inga capitata* Desv.	Fruit	Woody
	*Inga laurina* (Sw.) Willd.	Fruit	Woody
	*Inga subnuda* Salzm. ex Benth.	Fruit	Woody
	*Inga cinnamomea* Spruce ex Benth.	Fruit, Seed	Woody
	*Inga edulis* Mart.	Fruit	Woody
	*Inga marginata* Willd.	Fruit, Seed	Woody
	*Inga vulpina* Mart. ex Benth.	Fruit	Woody
	*Libidibia ferrea* (Mart. ex Tul.) L.P.Queiroz	Fruit, Seed	Woody
	*Bauhinia cheilantha* (Bong.) Steud.	Leaf, Seed	Woody
	*Senegalia bahiensis* (Benth.) Seigler & Ebinger	Fruit	Woody
Humiriaceae	*Vantanea bahiaensis* Cuatrec	Fruit	Woody
Lamiaceae	*Vitex megapotamica* (Spreng.) Moldenke	Stem	Woody
	*Vitex cymosa* Bertero ex Spreng.	Fruit, Flower	Woody
	*Ocimum carnosum* (Spreng.) Link & Otto ex Benth.	Leaf	Woody
Malpighiaceae	*Byrsonima cydoniifolia* A.Juss	Fruit	Woody
	*Bunchosia armeniaca* (Cav.) DC	Fruit	Woody
	*Byrsonima sericea* DC	Fruit	Woody
Malvaceae	*Guazuma ulmifolia* Lam.	Fruit	Woody
	*Sterculia striata* A.St.-Hil. & Naudin	Seed	Woody
	*Pachira aquatica* Aubl.	Seed	Woody
Marantaceae	*Maranta divaricata* Roscoe	Root, Leaf	Non-Woody
Melastomataceae	*Leandra australis* (Cham.) Cogn.	Fruit	Woody
	*Mouriri guianensis* Aubl.	Fruit	Woody
	*Mouriri pusa* Gardner	Fruit	Woody
	*Miconia albicans* (Sw.) Steud.	Fruit	Woody
Meliaceae	*Guarea macrophylla* Vahl	Fruit	Woody
Menispermaceae	*Abuta grandifolia* (Mart.) Sandwith	Fruit	Woody
Moraceae	*Maclura tinctoria* (L.) D.Don ex Steud.	Fruit	Woody
	*Brosimum glaziovii* Taub	Fruit	Woody
	*Ficus clusiifolia* Schott	Fruit	Woody
Myrtaceae	*Eugenia uniflora* L.	Fruit	Woody
	*Plinia peruviana* (Poir.) Govaerts	Fruit	Woody
	*Psidium cattleyanum* Sabine	Fruit	Woody
	*Plinia edulis* (Vell.) Sobral	Fruit	Woody
	*Eugenia punicifolia* (Kunth) DC	Fruit	Woody
	*Psidium laruotteanum* Cambess.	Fruit, Leaf	Woody
	*Psidium myrsinites* DC	Fruit	Woody
	*Psidium guineense* Sw	Fruit	Woody
	*Myrcia guianensis* (Aubl.) DC	Fruit	Woody
	*Eugenia candolleana* DC	Leaf	Woody
	*Campomanesia guazumifolia* (Cambess.) O.Berg	Fruit	Woody
	*Eugenia arenaria* Cambess.	Fruit	Woody
	*Eugenia pruniformis* Cambess.	Fruit	Woody
	*Neomitranthes obscura* (DC.) N.Silveira	Fruit	Woody
	*Campomanesia adamantium* (Cambess.) O.Berg	Fruit	Woody
	*Campomanesia guaviroba* (DC.) Kiaersk.	Fruit	Woody
	*Eugenia brasiliensis* Lam.	Fruit	Woody
	*Eugenia itaguahiensis* Nied	Fruit	Woody
	*Myrciaria glazioviana* (Kiaersk.) G.M.Barroso ex Sobral	Fruit	Woody
	*Plinia coronata* (Mattos) Mattos	Fruit	Woody
	*Psidium grandifolium* Mart. ex DC	Fruit	Woody
	*Myrciaria strigipes* O.Berg	Fruit	Woody
	*Eugenia pyriformis* Cambess.	Fruit	Woody
	*Psidium schenckianum* Kiaersk.	Fruit	Woody
Nymphaeaceae	*Victoria amazonica* (Poepp.) J.E.Sowerby	Seed	Non-Woody
Olacaceae	*Ximenia americana* L.	Fruit	Woody
Passifloraceae	*Passiflora foetida* L.	Fruit	Woody
	*Passiflora cincinnata* Mast	Seed	Woody
	*Passiflora misera* Kunth	Seed	Woody
	*Passiflora alata* Curtis	Fruit	Woody
	*Passiflora edulis* Sims	Fruit	Woody
	*Passiflora mucronata* Lam.	Fruit	Woody
	*Passiflora silvestris* Vell.	Fruit	Woody
	*Passiflora mediterranea* Vell.	Fruit	Woody
	*Passiflora amethystina* J.C.Mikan	Fruit	Woody
Plantaginaceae	*Plantago tomentosa* Lam.	Whole Plant	Non-Woody
Poaceae	*Oryza glumaepatula* Steud.	Seed	Non-Woody
	*Oryza latifolia* Desv.	Seed	Non-Woody
Polygonaceae	*Coccoloba parimensis* Benth.	Fruit	Woody
Portulacaceae	*Portulaca grandiflora* Hook.	Leaf	Non-woody
Primulaceae	*Myrsine umbellata* Mart.	Fruit	Woody
Rhamnaceae	*Sarcomphalus joazeiro* (Mart.) Hauenschild	Fruit	Woody
	*Rhamnidium elaeocarpum* Reissek	Fruit	Woody
	*Sarcomphalus undulatus* (Reissek) Hauenschild	Fruit	Woody
	*Condalia buxifolia* Reissek	Fruit	Woody
	*Scutia arenicola* (Casar.) Reissek	Fruit	Woody
Rosaceae	*Rubus sellowii* Cham. & Schltdl.	Fruit	Woody
	*Rubus brasiliensis* Mart.	Fruit	Woody
Rubiaceae	*Alibertia edulis* (Rich.) A.Rich	Fruit	Woody
	*Genipa americana* L.	Fruit	Woody
	*Tocoyena formosa* (Cham. & Schltdl.) K.Schum.	Fruit	Woody
	*Randia armata* (Sw.) DC	Fruit	Woody
Rutaceae	*Esenbeckia almawillia* Kaastra	Leaf	Woody
Sapindaceae	*Melicoccus lepidopetalus* Radlk.	Fruit	Woody
	*Talisia esculenta* (Cambess.) Radlk.	Fruit	Woody
	*Talisia macrophylla* (Mart.) Radlk.	Fruit	Woody
Sapotaceae	*Sideroxylon obtusifolium* (Roem. & Schult.) T.D.Penn	Fruit	Woody
	*Pouteria glomerata* (Miq.) Radlk.	Fruit	Woody
	*Chrysophyllum arenarium* Allemão	Fruit	Woody
	*Pouteria macrophylla* (Lam.) Eyma	Fruit	Woody
	*Pouteria caimito* (Ruiz & Pav.) Radlk.	Fruit	Woody
	*Micropholis venulosa* (Mart. & Eichler) Pierre	Fruit	Woody
Solanaceae	*Solanum agrarium* Sendtn	Fruit	Woody
	*Capsicum baccatum* L.	Fruit	Woody
	*Capsicum praetermissum* Heiser & P. G. Sm	Fruit	Woody
	*Solanum aculeatissimum* Jacq.	Fruit	Woody
	*Solanum americanum* Mill	Fruit, leaf	Non-woody
	*Solanum paniculatum* L.	Fruit	Woody
Talinaceae	*Talinum fruticosum* (L.) Juss	Leaf	Non-woody
	*Talinum paniculatum* (Jacq.) Gaertn	Leaf, stem	Non-woody
Typhaceae	*Typha domingensis* Pers.	Stem	Non-woody
Urticaceae	*Cecropia pachystachya* Trécul	Fruit	Woody
Vitaceae	*Clematicissus simsiana* (Schult. & Schult.f.) Lombardi	Underground Organ	Woody

**Fig. 2:**
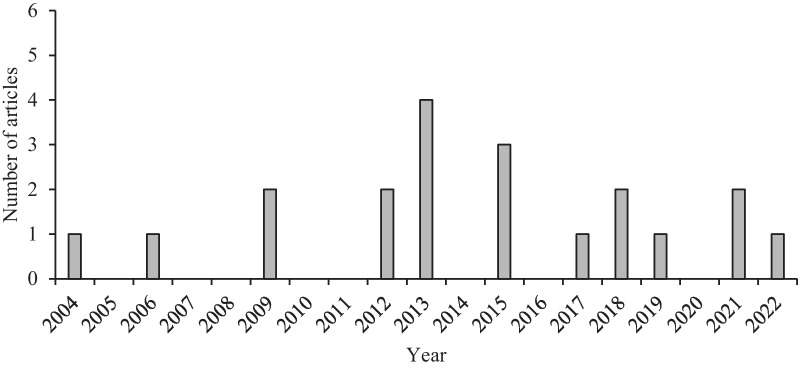
Number of articles per year

The distribution of studies per year is shown in Fig. 2. Three or more articles were found in the years 2013, 2015 and 2021, demonstrating a slight trend of increase in the number of works considered to present low or moderate risk of bias from 2013 onwards.

Most of the studies were conducted in the Northeastern and Southeastern regions of Brazil, representing more than 80% of the studies considered in this review. Regarding biomes, 95% of the studies were conducted in the Atlantic Forest and Caatinga. Studies conducted in rural areas predominated, corresponding to 75% of the total.

**Fig. 3:**
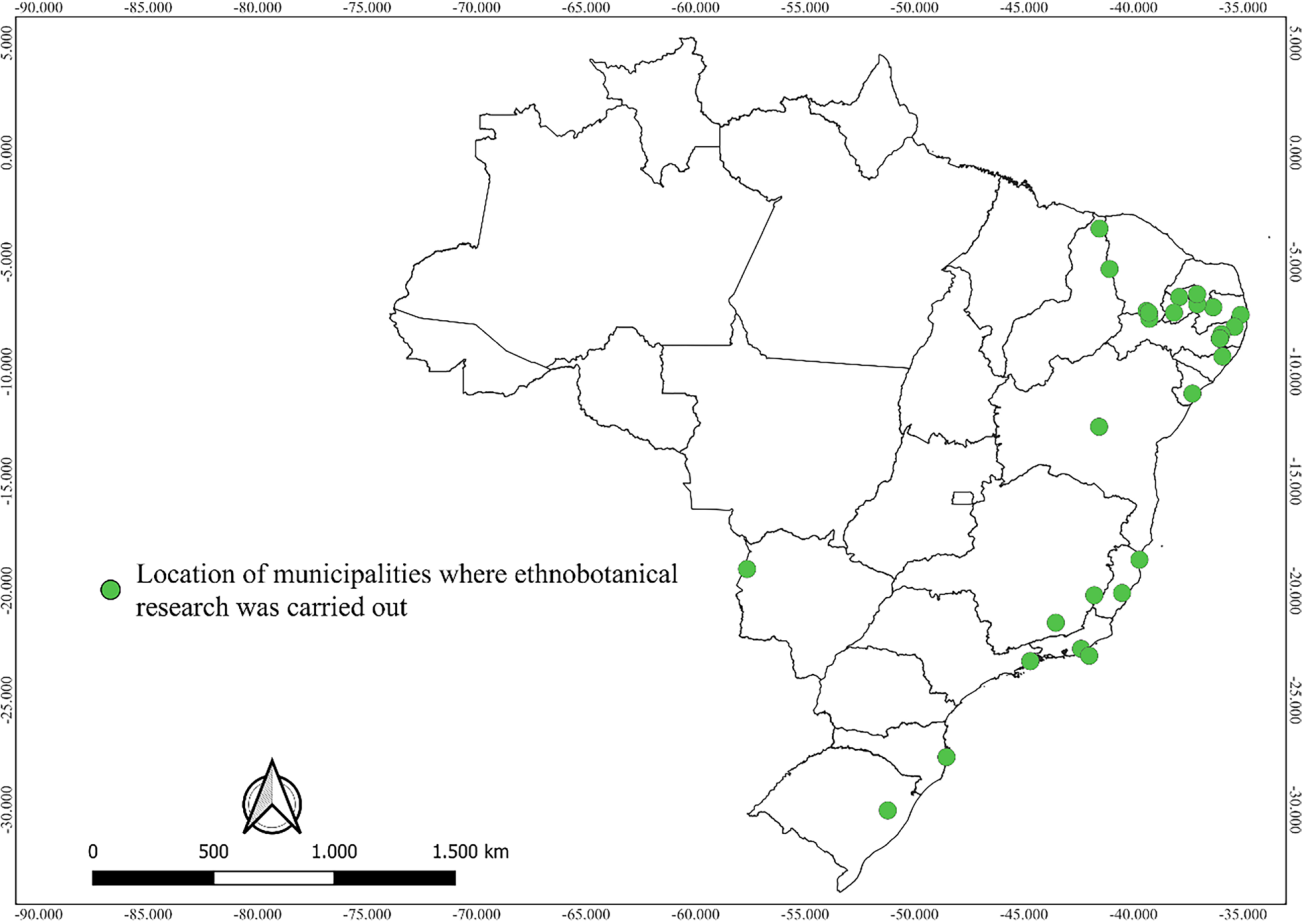
Distribution map of studies with an ethnobotanical approach, without a high risk of bias, conducted with wild food plants of Brazil

The map (Fig. 3) shows that there was a greater concentration of studies without a high risk of bias in the Northeast and Southeast regions of the country, more specifically in the Atlantic Forest and Caatinga biomes.

Regarding the distribution of species in the biomes, studies from the Atlantic Forest had greater species richness (53.6%), followed by Caatinga (28.4%), and Pantanal (18.0%).

**Fig. 4:**
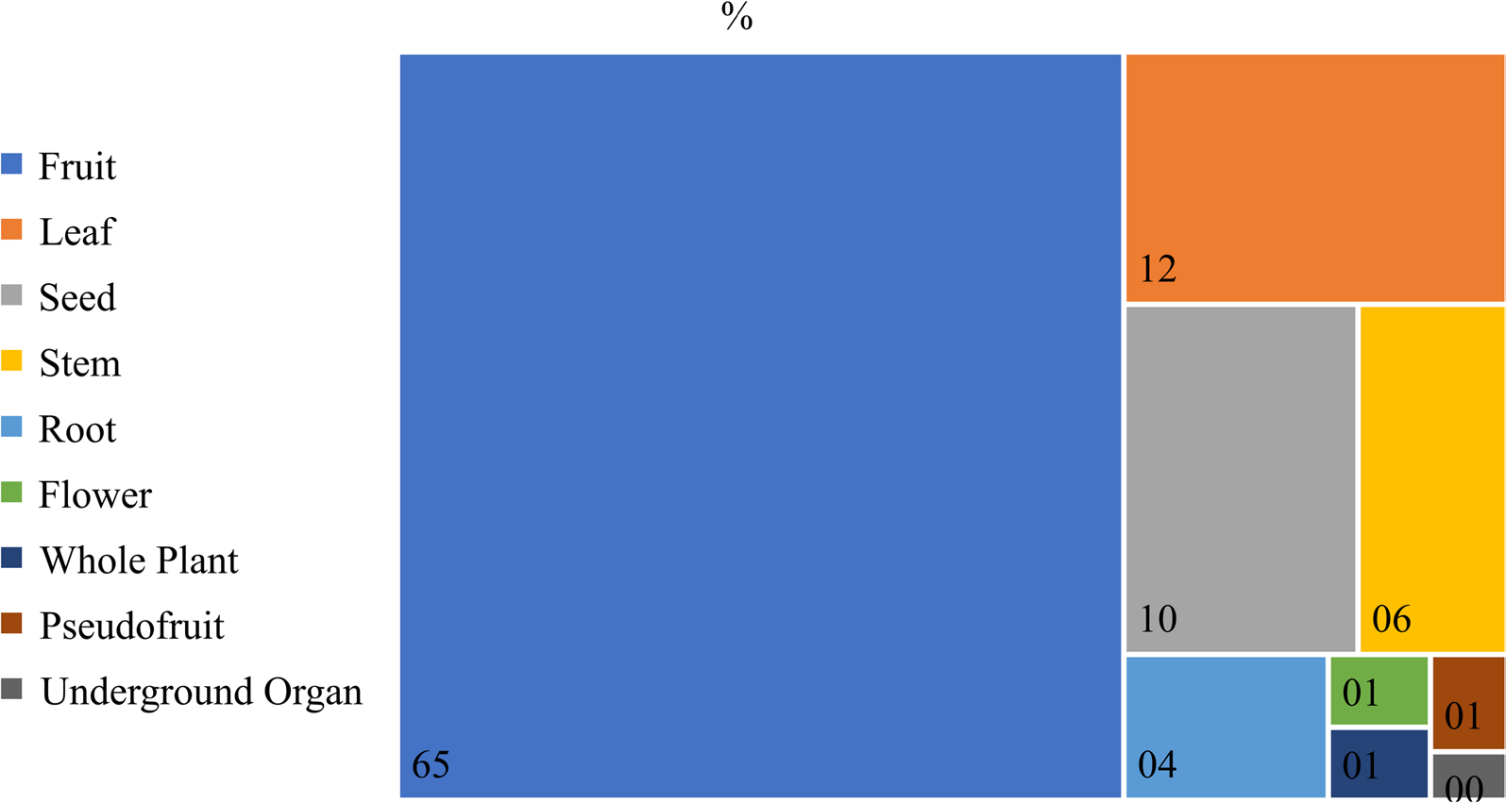
Proportion of use of parts of wild food plants in studies carried out in Brazil with an ethnobotanical approach

As for the parts used, there was a predominance of fruits, followed by leaves and seeds, which together represented 85.8% of the total parts (Fig. 4).

### Is the consumption of wild food plants by local Brazilian populations predominantly focused on reproductive parts?

**Fig. 5:**
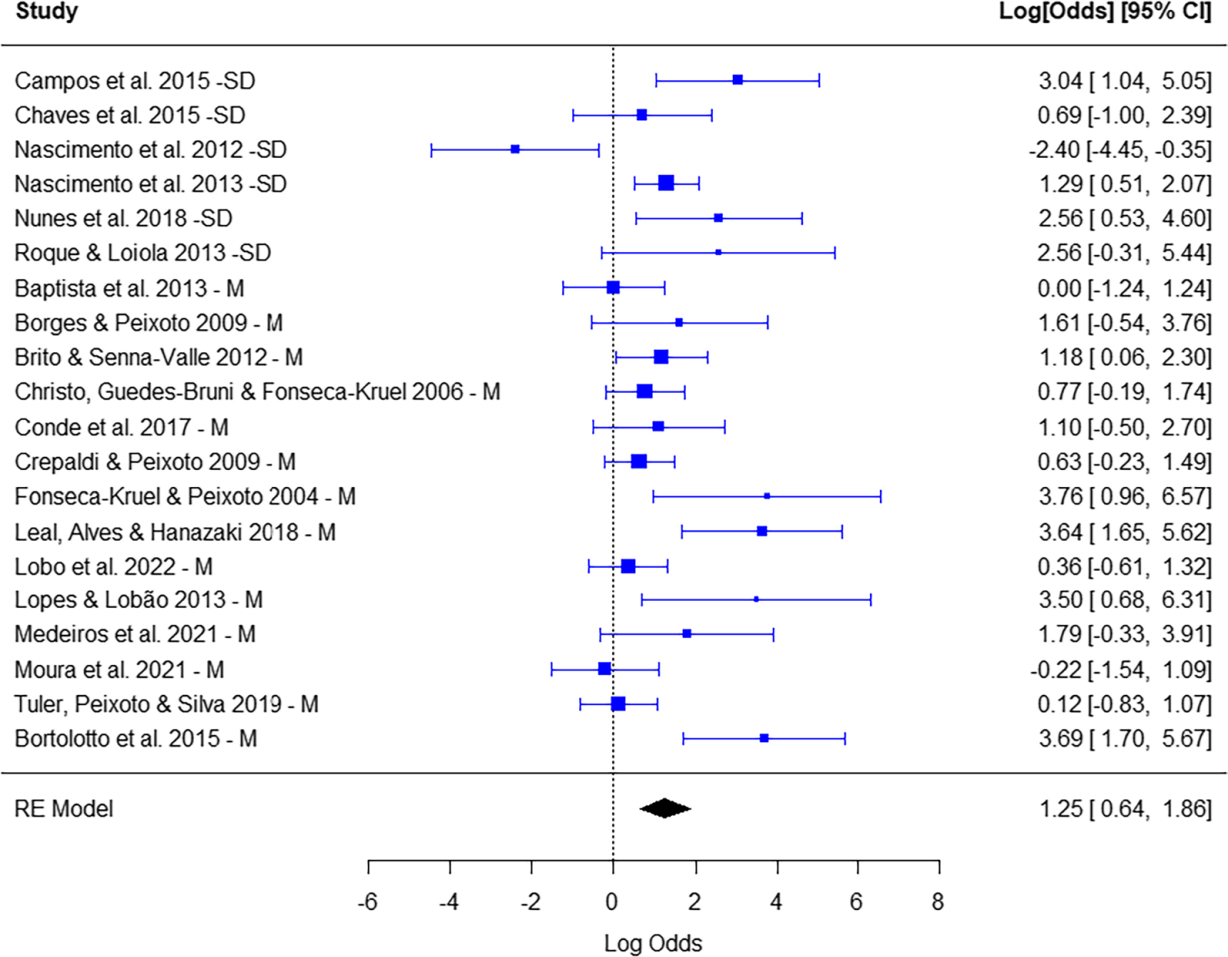
Number of species whose non-reproductive (left) *x* reproductive (right) parts were consumed in each study. Calculation of the effect size for each study (proportion of reproductive parts with logit transformation). Random effects model to identify patterns among studies and heterogeneity. The type of ecosystem—seasonally dry (SD) *x* moist (M)—was the moderator. CI—confidence interval

The consumption of reproductive parts predominated (flowers, fruits, pseudofruits and seeds) (summarized proportion = 1.25; CI (95%) = 0.64, 1.86; *p* < 0.0001). There was also heterogeneity, that is, although there was a general trend of predominance of consumption of reproductive parts, the studies yielded heterogeneous results (test statistics for the test of heterogeneity (*Q*) = 51,80; *p* < 0.0001). Finally, the type of ecosystem did not interfere with the proportion of reproductive parts (− 0.05; CI (95%) = − 1.47, 1.36; *p* > 0.05) (Fig. 5).

### Is the consumption of wild food plants by local Brazilian populations predominantly focused on destructive parts?

**Fig. 6:**
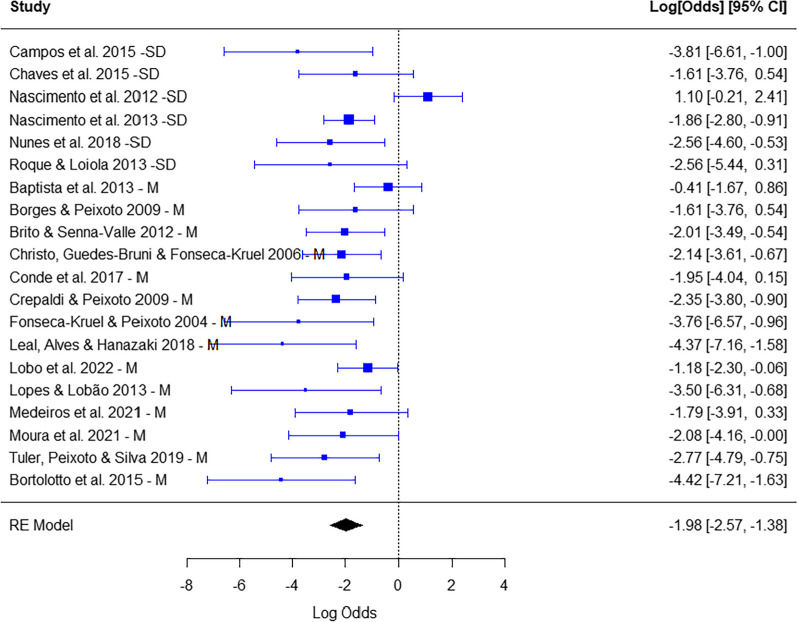
Number of species with consumption of non-destructive (left) *x* destructive (right) parts in each study. Calculation of effect size for each study (proportion of reproductive parts with logit transformation). Random effects model to identify patterns among studies and heterogeneity. The type of ecosystem—seasonally dry (SD) *x* moist (M)—was the moderator. CI—confidence interval

There was a predominance of consumption of non-destructive parts (leaves, flowers, fruits, pseudofruits and seeds) (summarized proportion = − 1.98; CI (95%) = − 2.57, − 1.38; *p* < 0.0001). There was heterogeneity, that is, although there was a general trend toward predominance of consumption of non-destructive, the studies yielded heterogeneous results (*Q* = 39,17; *p* < 0.0001). There was no interference of type of ecosystem in the proportion of non-destructive parts (0.65; CI (95%) = − 0.63, 1.92; *p* > 0.05) (Fig. 6).

### Is the consumption of wild food plants by local Brazilian populations predominantly focused on persistent parts?

**Fig. 7:**
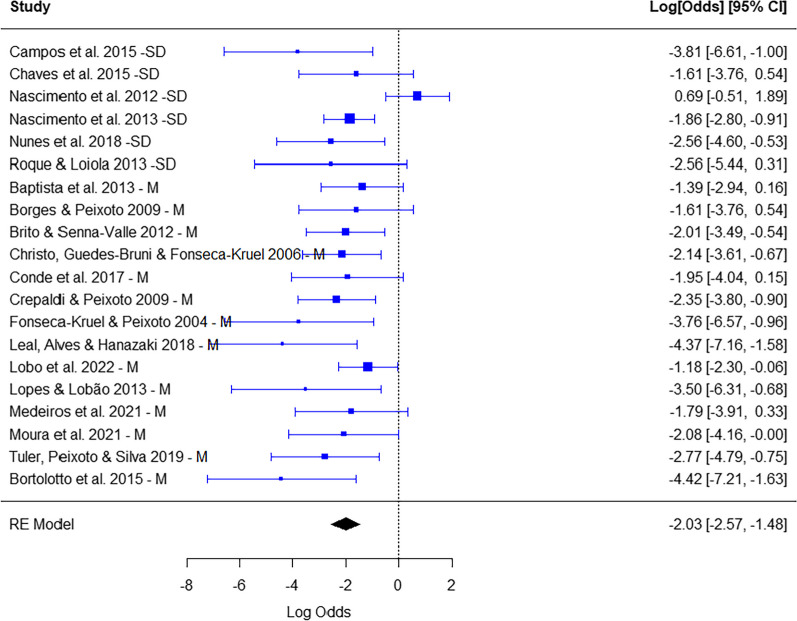
Number of species whose non-persistent (left) *x* persistent (right) parts were consumed in each study. Calculation of effect size for each study (proportion of reproductive parts with logit transformation). Random effects model to identify patterns among studies and heterogeneity. The type of ecosystem—seasonally dry (SD) *x* moist (M)—was the moderator. CI—confidence interval

There was a predominance of consumption of non-persistent parts (leaves, flowers, fruits, pseudofruits and seeds) (Summarized proportion = − 2.03; CI (95%) = − 2.57, − 1.48; *p* < 0.0001). There was heterogeneity, that is, although there was a general trend of predominance of non-persistent parts, the studies had heterogeneous results (*Q* = 32,62; *p* < 0.001). The type of ecosystem did not interfere with the proportion of non-persistent parts (0.72; CI (95%) = − 0.40, 1.86; *p* > 0.05) (Fig. 7).

### Is the consumption of wild food plants by local Brazilian populations predominantly focused on woody plants?

**Fig. 8:**
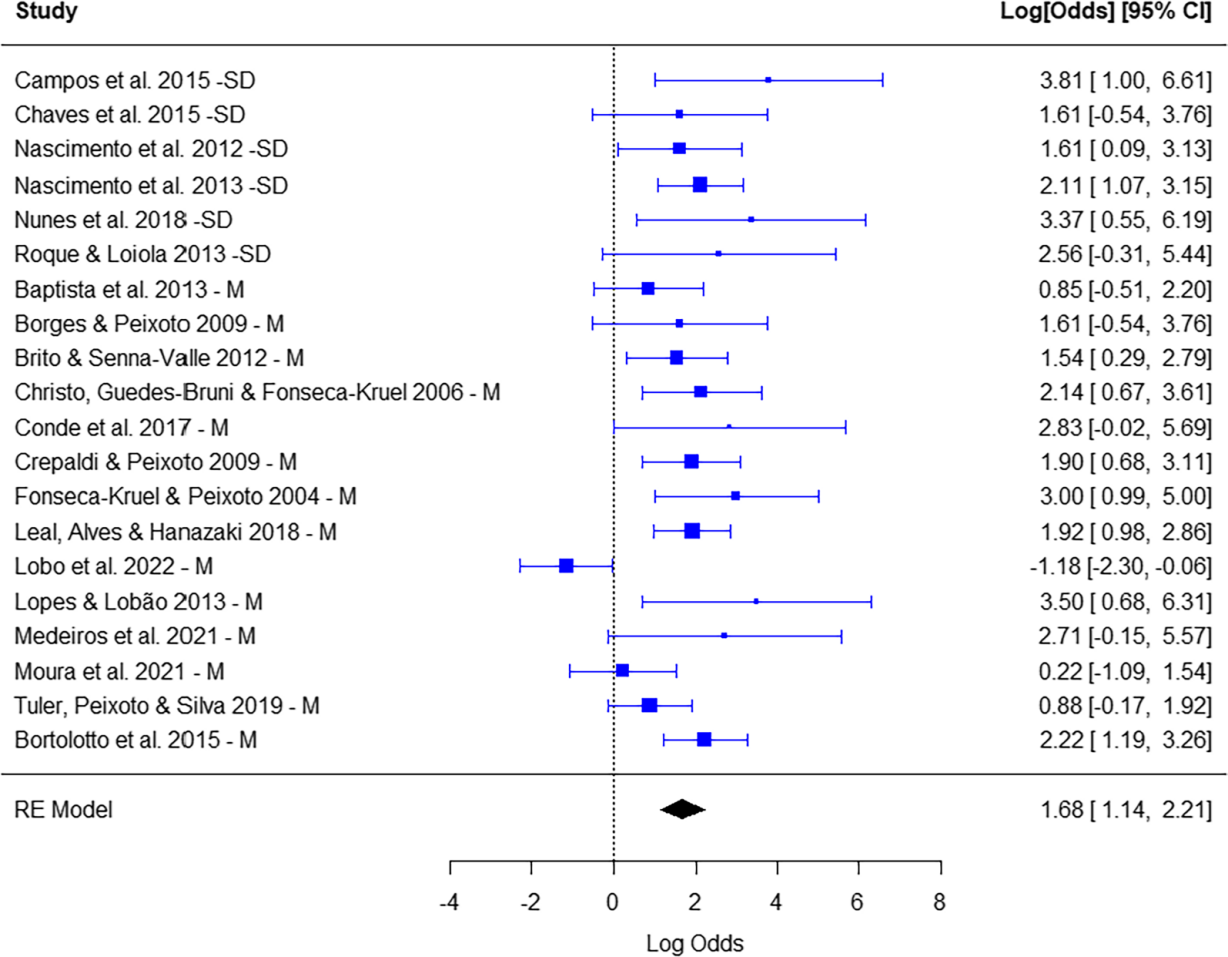
Number of non-woody (left) *x* woody (right) species whose parts were consumed in each study. Calculation of effect size for each study (proportion of reproductive parts with logit transformation). Random effects model to identify patterns among studies and heterogeneity. The type of ecosystem—seasonally dry (SD) *x* moist (M)—was the moderator. CI—confidence interval

There was a predominance of consumption of parts of woody plants (summarized proportion = 1.68; CI (95%) = 1.14, 2.21; *p* < 0.0001). There was heterogeneity, that is, although there was a general trend of predominance of woody plants, the studies had heterogeneous results (*Q* = 43,40; *p* > 0.05). The type of ecosystem did not interfere with the proportion of woody plants (0.80; CI (95%) = − 0.41, 2.02; *p* > 0.05) (Fig. 8).

## Discussion

### Temporal and spatial distribution of studies with wild food plants of Brazil

Ethnobotanical works carried out with wild food plants of Brazil without a high risk of bias were concentrated in two biomes: the Atlantic Forest and Caatinga. Only one study was performed in Pantanal. The predominance of the Atlantic Forest and Caatinga as study areas of works without a high risk of bias was also observed in a national-scale meta-analysis of patterns of use of medicinal plants [3]. Several factors may be underlying this trend, including issues related to the distribution and profile of research groups. A high number of ethnobiological studies have been conducted in ecosystems of Cerrado (savannas and seasonally dry forests), for example, but most of them have a qualitative nature and make use of theoretical samples that make sense for their objectives, but which are not suitable for meta-analyses.

Therefore, he absence of studies in the Amazon and Cerrado does not necessarily imply that these biomes are inadequately represented in terms of ethnobiological research efforts. It simply signifies that the epistemological orientation of these studies does not align with the criteria for inclusion in a meta-analysis. While we did not incorporate Amazonian studies into our research, there is evidence to suggest that, in certain aspects, their patterns of wild food plant consumption are not significantly different from those in other Brazilian biomes. For instance, a study conducted in the Amazon, which did not meet our inclusion criteria, gathered information from fieldwork in various communities and concluded that the consumption of greens in the region is low, as people tend to prefer wild fruits and tubers [41].

Regarding the temporal distribution of the studies, there was a slight increase in the number of studies without a high risk of bias starting in the year 2013. This may be related to the increasing number of courses and manuals about methods in this field of research that led to greater care in the selection of more robust samples. Furthermore, the studies of Kinupp & Barros in 2007 and 2008 [42, 43] and the further publication of the book “Plantas Alimentícias Não Convencionais” (unconventional food plants) in 2014 by Lorenzi and Kinupp [44] brought popularity to this topic and could also have helped to increase the number of research groups interest in studying unconventional or wild food plants.

Eliminating sampling problems from quantitative ethnobiological studies is fundamental from the point of view of biodiversity conservation because information with sampling problems and biased results may have an influence on decisions and actions. Biased results include, for example, misleading clues about the conservation status of some plants, the identity of the most popular plant species and the strategies necessary for management [16].

The elimination of study bias can be achieved through a good sample design, which means having a sample that accurately reflects the entire population while respecting the margin of error and confidence interval. However, not only does this ensure representativeness, but it is also necessary to adhere to the principles of randomness, thereby avoiding the influence of specific groups, particularly in the context of quantitative research [16]. It is also important to identify the plant material, specify that the material was identified by comparing voucher specimens or consulting experts, and provide a complete list of species [3].

### Patterns related to the parts used and the seasonality factor

Our findings point to a high predominance of use of fruits of food plants native to Brazil, which in turn influences the predominance of use of reproductive, non-persistent, and non-destructive parts. The predominance of fruits was also found in other studies at different scales, such as in the Yi peoples in China, where fruits were the most used part, followed by roots and shoots [45], and in the Kaski district (Nepal), where fruits were also the most used part, followed by young shoots [46].

However, leaves are the most consumed parts of wild food plants in different regions of the world, such as among the Vasavas in India [47], the Mapuche in Argentina [48], among ethnic minorities in Yunnan, China [9] and in two valleys of the Qinling mountains, Shaanxi, China [49].

The reasons for regional or national differences in use patterns may be plenty. First, it may have to do with the availability of edible parts in each region. For example, tropical ecosystems are more likely to produce fleshy fruits [50]. In many contexts, fruits may be preferred over green vegetables, considering that leaves are more likely to have bitter tastes, which are usually more avoided due to evolutionary processes (most toxic products in nature have bitter teste) [51]. Therefore, in contexts where food is not scare (e.g., high presence of fruits and other plant parts other than leaves), people would avoid using green parts of plants, in a process that is sometimes called herbophobia [52].

Moreover, in some tropical and subtropical regions there is a higher occurrence of thick leathery leaves [49], which are often not considered the most appropriate leaves for edible purposes. Those ecological patterns probably influence a higher use of fruits when compared to leaves in the tropics, but the existence of such prevalence needs to be further investigated.

On the other hand, cultural aspects may also play a significant role in shaping the consumption of leaves from wild food plants. For instance, in many local Chinese communities, the consumption of green leaves is more substantial compared to many other regions worldwide, making them some of the most herbophilous communities globally [52, 53]. The cultural forces that drive this high consumption of greens can be observed even in their language, as many plant species have popular names containing the word ‘cai’ (meaning ‘vegetable’), which encodes the edible nature of these greens in the language [53]. Furthermore, the cultural importance of greens is evident in the fact that these communities continue to practice traditions that have been lost in many parts of the world, such as the drying of wild vegetables for winter storage [53]

The patterns of preference for specific plant parts differ between food and medicinal plants in the Brazilian context: while fruits are preferred in the first group, leaves prevail in the second [3]. The preference for fruits for food purposes may be associated with the fact that this part has constituents with greater nutritional quality for consumers. In turn, leaves have a higher concentration of therapeutic agents, what explains why they are more frequently used in medicinal preparations.

Among the plant parts used for food, the parts classified as reproductive, non-persistent, non-destructive and parts of woody plants prevailed. However, the type of ecosystem had no influence on the use patterns, that is, local populations in seasonally dry and moist environments did not differ in terms of use patterns. Thus, it is possible that people’s preferences are not influenced by seasonality, but by other environmental conditions and factors. In the case of persistence and habit, the present study did not support the seasonality hypothesis, contrary to works focused on medicinal plants [3, 15].

An important factor that explains these differences is that although persistent parts of plants are important sources of compounds with medicinal properties, they are often not suitable for consumption as food, given that a small number of species have tubers or similar parts, or even stems with food potential.

The fact that wild food plants were not the food base of the groups in most studies may also explain the absence of strategies to secure the access to these resources in seasonally dry environments. When other products meet dietary needs, the spatial or temporal availability of wild food plants may lose relevance in relation to other variables, such as flavor [54].

Thus, considering the complementary role of wild food plants in the diet of most Brazilian populations and from the point of view of the socio-ecological theory of maximization, even if fruits and other non-persistent parts are not available year round, other variables may confer a great advantage to these resources to the point that availability becomes secondary [55].

From the point of view of conservation, the predominance of the use of non-destructive plant parts, especially fruits, places food use among factors with a low potential impact, corroborating the literature regarding non-timber forest products [12]. Such lower potential impact favors the stimulation of sustainable use and even the popularization of wild food plants as a strategy for food diversification and for increasing food and nutritional security. However, it is necessary to consider that, despite the lower impact in comparison with, for example, timber products, the use of wild food plants requires management strategies aimed at the preservation of plant populations. In this sense, the predominance of use of reproductive plant parts indicates the need for strategies to monitor the recruitment of new individuals, which can be operationalized through participatory management (or co-management). Participatory management is understood as the cooperation between government agencies, traditional local communities, resource users, as well as non-governmental organizations and other stakeholders, sharing the management and responsibility for an area or a set of resources [56]. In the context of native wild food plant use in Brazil, we recommend training local harvesters to conduct both qualitative and quantitative monitoring of seedlings. Their constant presence in the harvesting areas will enable more frequent monitoring. Additionally, the entire process should not be top-down planned, as communities should also have a say in determining the main purposes and objectives of the conservation strategies.

## Conclusion

The concentration of studies in the Northeast, Southeast and South regions of Brazil and in the Atlantic Forest and Caatinga biomes points to the need for a greater effort in terms of quantitative ethnobotanical research in other regions and biomes so as to contribute to a more effective search for patterns of use of wild food plants in the country.

The predominance of fruits and plant parts classified as reproductive, non-persistent and non-destructive points to the high potential for implementation of sustainable management strategies aimed at these plants in the country and also suggests the possibility of popularization and expansion of their consumption. However, for this, there needs to be a solid participatory monitoring, especially of the recruitment of new individuals, aiming at controlling the amount of fruits that can be gathered in order to maintain the local stocks of wild food plants.

The lack of differences in use patterns between seasonally dry and moist ecosystems demonstrates that the influence of seasonality on plant selection observed in previous studies with medicinal plants does not necessarily apply to food products. This is possibly due to the inadequacy of persistent parts for food use, the fact that wild species are not the food base of populations in most cases, and the fact that other variables possibly compensate for the lower temporal availability of non-persistent resources, such as their taste, which are often more attractive than those from other plant parts.

This research has some evident limitations. Since we are dealing with secondary data and each study addresses different aspects of wild food plant use, we could only focus on some quantitative aspects of such use. Therefore, we ended up missing important issues concerning, for example, cultural differences and how they can influence wild food plant use. For this purpose, an ideal research design would have to consider a framework in which socioeconomic and cultural information could be systematically and uniformly collected in different Brazilian communities.

### Supplementary Information

**Additional file 1. **Criteria for assessing bias risk.

**Additional file 2. **General information on all 79 articles that went through the inclusion/exclusion process.

## Data Availability

https://drive.google.com/drive/folders/1Kh_1zkGGhD7xZtEwIzMektutswFA59jO?usp=share_link.

## Abbreviations

PLO: Logit-transformed proportion
SD: Seasonally dry
M: Moist
*Q*: Test statistics for the test of heterogeneity
CI: Confidence interval
