# Supplementary material for: Patterns of use of wild food plants by Brazilian local communities: systematic review and meta-analysis
Source: J Ethnobiol Ethnomed. 2023 Oct 25;19:47. doi: 10.1186/s13002-023-00619-y (PMC10601232; doi:10.1186/s13002-023-00619-y)
Supplement: Supplementary file 1 — Additional file 1. Criteria for assessing bias risk. [file 13002_2023_619_MOESM1_ESM.docx]

**Additional file 1**

Establishing the risk of bias according to Medeiros PM, Ladio AH, Albuquerque UP. Sampling problems in Brazilian research: A critical evaluation of studies on medicinal plants. Revista Brasileira de Farmacognosia. 2014;24:103–9.

**1) When sample is extracted from the total number of people or from an age interval**

**Low**

a) When the sample size (N) reaches the universe (U)

b) When N is representative of U, with sample randomness and considering a margin of error of up to 5%

c) When N is at least 80% of U, considering that some respondents may refuse to participate of interviews or may not be in their

household even after successive trials.

**Moderate**

a) When N is extracted from U, with sample randomness and a margin of error higher than 5% and lower than 10%.

b) When N is at least 80% of the value is needed for representativeness, considering a margin of error of up to 5%.

c) When N could be considered to be representative of U (with a margin of error of up to 10%) if only the numbers are considered, but in

situations in which sample is occasional or when there is no specificity about randomness.

**High**

a) When N is extracted from U with a margin of error higher than 10%.

b) When N is less than 80% of the value is necessary for representativeness, considering a margin of error of up to 5%.

c) When there is no information about the universe (U), or when there is no information about the sample (N).

**2) When sample is based on the heads of family (one or two per household)**

**Low**

a) When all heads of family were interviewed.

b) When a representative number of heads of family were interviewed, with sample randomness and a margin of error up to 5%.

c) When N is at least 80% of the heads of family

**Moderate**

a) When N is extracted from the universe (U) of family chiefs, with sample randomness and a margin of error higher than 5% and lower

than 10%.

b) When N is at least 80% of the value necessary for the representativeness of the heads of family, considering a margin of error of up to 5%.

c) When N could be considered to be representative of the number of heads of family (with a margin of error of up to 10%), if only the

numbers are considered, but in situations which sample is occasional or when there is no specificity about randomness.

**High**

a) When N is extracted from the universe (U) of heads of family with a margin of error of up to 10%

b) When N is less than 80% of the value needed for representativeness of the family chiefs, considering a margin of error of up to 5%.

c) When there is no information about the number of heads of family, or when there is no information about the sample (N).

**3) When sample is based on households**

**Low**

a) When one member of each household was interviewed.

b) When a representative number of households had one of its members interviewed, with sample randomness and a margin of error up to 5%.

c) When N is at least 80% of the households.

**Moderate**

a) When N is extracted from the universe (U) of households, with sample randomness and a margin of error higher than 5% and lower than 10%.

b) When N is at least 80% of the value necessary for sample representativeness, considering a margin of error of 5%.

c) When N could be considered to be representative of the households (with a margin of error of up to 10%) if only the numbers are

considered, but in situations in which sample is occasional or when there is no specificity about randomness.

**High**

a) When N is extracted from the universe (U) of households with a margin of error higher than 10%.

b) When N is less than 80% of the value which is necessary for household representativeness, considering a margin of error of up to 5%.

c) When there is no information about the number of households (U), or when there is no information about the sample (N).

**4) When sample is intentional, focusing on a group of interest (e.g. midwives, healers, local specialists)**

**Low**

a) When sample corresponds to the totality of the specific group.

b) When sample is representative of the specific group, with sample randomness and a margin of error or up to 5%).

c) When sample is at least 80% of the specific group.

d) In cases of local specialists, when the snowball technique is used and there is an indication of the total number of dwellers.

e) In cases of local specialists, when they are selected based on clear and well established criteria.

**Moderate**

a) When N is extracted from the universe (U) of the specific group, with sample randomness and a margin of error higher than 5% and

lower than 10%.

b) When N is at least 80% of the value necessary of representativeness of the specific group, considering a margin of error of up to 5%.

c) When N could be considered to be representative of the specific group (with a margin of error of up to 10%) if only the numbers are

considered, but in situations which sample is occasional or when there is no specificity about randomness.

d) In cases of local specialists, when there is no indication of the universe (U), but the snowball technique is applied to select the key

respondents.

**High**

a) When N is extracted from the universe (U) of the specific group with a margin of error higher than 10%.

b) When N is less than 80% of the value which is necessary for representativeness of the specific group, considering a margin of error of up to 5%.

c) When there is no information about the specific group (U), or when there is no information about the sample (N), except for the use of

the snowball technique, when there is no information about the U.

d) In cases of local specialists, when they are selected based on arbitrary or obscure criteria.

**5) When rarefaction curves are used**

**Low**

a) When there is information about N and U and when the rarefaction curve stabilizes, regardless of sample representativeness and the

criteria for respondent selection.

Moderate

a) When there is no information about U, but the rarefaction curve stabilizes

b) When there is no information about N and U and when the rarefaction curve gets close to stabilization.

**High**

a) When there is no information about N, regardless of the rarefaction curve behavior.

b) When the rarefaction curve gets far from stabilization.

c) When the study claims to have performed a rarefaction curve, but does not exhibit its results and does not affirm that there was

stabilization.

**6) When participatory methods are used**

**Low**

a) When the number of participants corresponds to a representative amount of the population or specific group (with a margin of error of

up to 5%, but not considering the precepts of randomness, that mostly does not apply to participatory methods).

**Moderate**

a) When the number of participants is not representative of the population or specific group.

b) When there is no information about the universe (population as a whole or specific group), but there is information about the number of

participants.

**High**

a) When there is no information about the number of participants.

**7) Diffuse selection criteria**

**High**

a) When there is no information about N or U.

b) When there are various diffuse criteria for selecting the same sample.
