# Supplementary material for: Patterns of use of wild food plants by Brazilian local communities: systematic review and meta-analysis
Source: J Ethnobiol Ethnomed. 2023 Oct 25;19:47. doi: 10.1186/s13002-023-00619-y (PMC10601232; doi:10.1186/s13002-023-00619-y)
Supplement: Supplementary file 2 — Additional file 2. General information on all 79 articles that went through the inclusion/exclusion process. [file 13002_2023_619_MOESM2_ESM.docx]

**Additional file 2**

General information on all 79 articles that went through the inclusion/exclusion process

| **Author** | **Year** | **Periodic** | **JCR (2022)** | | **Ecosystem** | **Community type** | **Taxon** |
| --- | --- | --- | --- | --- | --- | --- | --- |
| Cruz et al. [1] | 2014 | Journal of Ethnobiology and Ethnomedicine | 3.6 | Caatinga | | Rural | Varied |
| Santos et al. [2] | 2009 | Economic Botany | 2.6 | Caatinga | | Rural | Varied |
| Moura et al. [3] | 2021 | Waste and Biomass Valorization | 3.2 | Atlantic Forest | | Artisanal fishermen | Varied |
| Borges & Peixoto [4] | 2009 | Acta Botanica Brasilica | 1.1 | Atlantic Forest | | Caiçaras¹ | Varied |
| Lucena et al. [5] | 2013 | Anais da Academia Brasileira de Ciências | N/i | Caatinga | | Rural | Varied |
| Barreira et al. [6] | 2015 | Revista Brasileira de Plantas Medicinais | N/i | Atlantic Forest | | Rural | Varied |
| Lima et al. [7] | 2012 | Acta Botanica Brasilica | 1.1 | Cerrado | | Geraizeiros | Varied |
| Brito & Senna-Valle [8] | 2012 | Acta Botanica Brasilica | 1.1 | Atlantic Forest | | Caiçaras | Varied |
| Araújo & Lopes [9] | 2012 | Biodiversity and Conservation | 3.4 | Amazônia | | Farmers | Arecaceae |
| Campos et al. [10] | 2015 | Journal of Arid Environments | 2.7 | Caatinga | | Extractivists | Varied |
| Santos & Andrade [11] | 2020 | Rodriguésia | N/i | Atlantic Forest | | Urban | Varied |
| Lobo et al. [12] | 2022 | Boletín Latinoamericano y del Caribe de Plantas Medicinales y Aromáticas | N/i | Atlantic Forest | | Gypsies | Varied |
| Silva & Andrade [13] | 2005 | Acta Botanica Brasilica | 1.1 | Atlantic Forest | | Rural | Varied |
| Silva et al. [14] | 2014 | Journal of Ethnobiology and Ethnomedicine | 3.6 | Caatinga | | Farmers | Varied |
| Mazon et al. [15] | 2020 | Food Science and Technology | 2.6 | Atlantic Forest | | Urban | Cactaceae |
| Souza et al. [16] | 2018 | Anais da Academia Brasileira de Ciências | N/i | Atlantic Forest | | Farmers | Varied |
| Bortolotto et al. [17] | 2015 | Journal of Ethnobiology and Ethnomedicine | 3.6 | Pantanal | | Rural | Varied |
| Nascimento et al. [18] | 2013 | Ecology of Food and Nutrition | 1.8 | Caatinga | | Rural | Varied |
| Cruz et al. [19] | 2013 | Journal of Ethnobiology and Ethnomedicine | 3.6 | Caatinga | | Rural | Varied |
| Leal et al. [20] | 2018 | Journal of Ethnobiology and Ethnomedicine | 3.6 | Atlantic Forest | | Rural | Varied |
| Nunes et al. [21] | 2018 | Journal of Ethnobiology and Ethnomedicine | 3.6 | Caatinga | | Rural | Varied |
| Gomes et al. [22] | 2020 | Ethnobiology and Conservation | 1.4 | Atlantic Forest | | Rural | Varied |
| Conde et al. [23] | 2017 | PLoS One | 3.7 | Atlantic Forest | | Quilombola² | Varied |
| Medeiros et al. [24] | 2021 | Scientifc Reports | 4.6 | Atlantic Forest | | Farmers | Varied |
| Rodrigues et al. [25] | 2020 | Journal of Ethnobiology and Ethnomedicine | 3.6 | Atlantic Forest | | Quilombola | Varied |
| Carneiro et al. [26] | 2010 | Acta Botanica Brasilica | 1.1 | Amazônia | | Artisanal fishermen | Varied |
| Pedrosa et al. [27] | 2021 | Environment, Development and Sustainability | 4.9 | Caatinga | | Rural | Varied |
| Chaves et al. [28] | 2015 | Revista Espacios | N/i | Caatinga | | Rural | Varied |
| Lucena et al. [29] | 2012 | Journal of Environmental Management | 8.7 | Caatinga | | Rural | Varied |
| Luijk et al. [30] | 2021 | Journal of Ethnobiology and Ethnomedicine | 3.6 | Atlantic Forest | | Rural | Varied |
| Tuler et al. [31] | 2019 | Rodriguésia | N/i | Atlantic Forest | | Farmers | Varied |
| Cunha et al. [32] | 2020 | Journal of Ethnobiology and Ethnomedicine | 3.6 | Atlantic Forest | | Urban | Varied |
| Lucena et al. [33] | 2013 | Journal of Ethnobiology and Ethnomedicine | 3.6 | Caatinga | | Rural | Cactaceae |
| Alarcón & Peixoto [34] | 2008 | Economic Botany | 2.6 | Amazônia | | Cablocos³ | Varied |
| Lucena et al. [35] | 2007 | Environmental Monitoring and Assessment | 3.0 | Caatinga | | Rural | Varied |
| Trotta et al. [36] | 2012 | Revista de estudos ambientais | N/i | Atlantic Forest | | Urban | Varied |
| Santos et al.[37] | 2013 | Revista Brasileira de Agroecologia | N/i | Atlantic Forest | | Farmers | Varied |
| Soldati et al. [38] | 2012 | Sitientibus série Ciências Biológicas | N/i | Atlantic Forest | | Farmers | Varied |
| Silva et al. [39] | 2014 | Boletim do Museu de Biologia Mello Leitão | N/i | Caatinga | | Rural | Varied |
| Miranda & Hanazaki [40] | 2007 | Acta Botanica Brasilica | 1.1 | Atlantic Forest | | Caiçaras e Açorianos⁴ | Varied |
| Rufino et al. [41] | 2008 | Acta Botanica Brasilica | 1.1 | Caatinga | | Rural | Arecaceae |
| Lucena et al. [42] | 2012 | Revista Biotemas | N/i | Caatinga | | Rural | Cactaceae |
| Strachulski & Floriani [43] | 2013 | Revista Geografar | N/i | Atlantic Forest | | Rural | Varied |
| Florentino et al. [44] | 2007 | Acta Botanica Brasilica | 1.1 | Caatinga | | Rural | Varied |
| Gandolfo & Hanazaki [45] | 2014 | Urban Ecosystems | 2.9 | Atlantic Forest | | Rural | Varied |
| Althaus-Ottmann et al. [46] | 2011 | Revista Brasileira de Biociências | N/i | Atlantic Forest | | Urban | Varied |
| Hanazaki et al. [47] | 2000 | Biodiversity and Conservation | 3.4 | Atlantic Forest | | Caiçaras | Varied |
| Ribeiro et al. [48] | 2014 | Ethnobotany Research & Applications | N/i | Caatinga | | Rural | Varied |
| Melo et al. [49] | 2008 | Rodriguésia | N/i | Atlantic Forest | | Artisanal fishermen | Varied |
| Alves et al. [50] | 2014 | Boletim do Museu de Biologia Mello Leitão | N/i | Caatinga | | Rural | Varied |
| Silva et al. [51] | 2007 | Journal of Ethnobiology | 2.9 | Amazônia | | Rural | Varied |
| Fernandes et al. [52] | 2014 | Rodriguésia | N/i | Atlantic Forest | | Farmers | Leguminosae |
| Gandolfo & Hanazaki [53] | 2011 | Acta Botanica Brasilica | 1.1 | Atlantic Forest | | Natives | Varied |
| Lopes & Lobão [54] | 2013 | Boletim do Museu de Biologia Mello Leitão | N/i | Atlantic Forest | | Artisanal fishermen | Varied |
| Fonseca-Kruel & Peixoto [55] | 2004 | Acta Botanica Brasilica | 1.1 | Atlantic Forest | | Artisanal fishermen | Varied |
| Miranda et al. [56] | 2011 | Rodriguésia | N/i | Atlantic Forest | | Caiçaras | Varied |
| Nascimento et al. [57] | 2012 | Economic Botany | 2.6 | Caatinga | | Rural | Varied |
| Poderoso et al. [58] | 2012 | Ethnobiology and Conservation | 1.4 | Atlantic Forest | | Rural | Varied |
| Filho et al. [59] | 2018 | Revista Fitos | N/i | Atlantic Forest e Caatinga | | Urban | Cactaceae |
| Zuchiwschi et al. [60] | 2010 | Acta Botanica Brasilica | 1.1 | Atlantic Forest | | Farmers | Varied |
| Lucena et al. [61] | 2008 | Ethnobotany Research & Applications | N/i | Caatinga | | Rural | Varied |
| Pilla & Amorozo [62] | 2009 | Acta Botanica Brasilica | 1.1 | Atlantic Forest | | Farmers | Varied |
| Roque & Loiola [63] | 2013 | Revista Caatinga | 0.9 | Caatinga | | Rural | Varied |
| Cunha & Albuquerque [64] | 2006 | Environmental Monitoring and Assessment | 3.0 | Atlantic Forest | | Rural | Varied |
| Eichemberg et al. [65] | 2009 | Acta Botanica Brasilica | 1.1 | Atlantic Forest | | Urban | Varied |
| Albuquerque et al. [66] | 2005 | Journal of Arid Environments | 2.7 | Caatinga | | Rural | Varied |
| Santos et al. [67] | 2014 | Economic Botany | 2.6 | Caatinga | | Rural | Varied |
| Baptista et al. [68] | 2013 | Journal of Ethnobiology and Ethnomedicine | 3.6 | Atlantic Forest | | Artisanal fishermen | Varied |
| Oliveira et al. [69] | 2020 | Ethnoscientia | N/i | Amazônia | | Indigenous | Varied |
| Santos et al. [70] | 2009 | Economic Botany | 2.6 | Atlantic Forest | | Rural | Myrtaceae |
| Neto et al. [71] | 2010 | Economic Botany | 2.6 | Caatinga | | Farmers | Anacardiaceae |
| Arévalo-Marín et al. [72] | 2015 | Ethnobotany Research & Applications | N/i | Caatinga | | Rural | Varied |
| Pedrosa et al. [73] | 2020 | Ethnobiology and Conservation | 1.4 | Caatinga | | Rural | Cactaceae |
| Crepaldi & Peixoto [74] | 2010 | Biodiversity and Conservation | 3.4 | Atlantic Forest | | Quilombola | Varied |
| Giraldi & Hanazaki [75] | 2014 | Human Ecology Review | 0.9 | Atlantic Forest | | Caiçaras | Varied |
| Milanesi et al. [76] | 2013 | Journal of Ethnobiology and Ethnomedicine | 3.6 | Atlantic Forest | | Rural | Varied |
| Christo et al. [77] | 2006 | Rodriguésia | N/i | Atlantic Forest | | Rural | Varied |
| Lucena et al. [78] | 2012 | Revista de Biologia e Farmácia | N/i | Caatinga | | Rural | Cactaceae |
| Guerra et al. [79] | 2012 | Revista de Biologia e Farmácia | N/i | Caatinga | | Rural | Varied |

N/i – No information

¹Traditional inhabitants of the coast of Southeastern Brazil;

² Descendants of Afro-Brazilian runaway slaves living in hideouts up-country cVarieded Quilombos

³ Traditional non-indigenous communities in the Amazon

⁴ Coming from the Azores archipelago, colonized by Portugal

**References**

1. Cruz MP, Medeiros PM, Sarmiento-Combariza I, Peroni N, Albuquerque UP. “I eat the manofê so it is not forgotten”: local perceptions and consumption of native wild edible plants from seasonal dry forests in Brazil. J Ethnobiol Ethnomed [Internet]. 2014;10:45. Available from: https://ethnobiomed.biomedcentral.com/articles/10.1186/1746-4269-10-45

2. Santos LL dos, Ramos MA, da Silva SI, de Sales MF, de Albuquerque UP. Caatinga Ethnobotany: Anthropogenic Landscape Modification and Useful Species in Brazil’s Semi-Arid Northeast. Econ Bot [Internet]. 2009;63:363–74. Available from: http://link.springer.com/10.1007/s12231-009-9094-3

3. Moura IO, Santana CC, Lourenço YRF, Souza MF, Silva ARST, Dolabella SS, et al. Chemical Characterization, Antioxidant Activity and Cytotoxicity of the Unconventional Food Plants: Sweet Potato (Ipomoea batatas (L.) Lam.) Leaf, Major Gomes (Talinum paniculatum (Jacq.) Gaertn.) and Caruru (Amaranthus deflexus L.). Waste Biomass Valorization [Internet]. 2021;12:2407–31. Available from: https://link.springer.com/10.1007/s12649-020-01186-z

4. Borges R, Peixoto AL. Conhecimento e uso de plantas em uma comunidade caiçara do litoral sul do Estado do Rio de Janeiro, Brasil. Acta Bot Brasilica [Internet]. 2009;23:769–79. Available from: http://www.scielo.br/scielo.php?script=sci_arttext&pid=S0102-33062009000300017&lng=pt&tlng=pt

5. Lucena RFP de, Lucena CM, Araújo EL, Alves ÂGC, Albuquerque UP de. Conservation priorities of useful plants from different techniques of collection and analysis of ethnobotanical data. An Acad Bras Cienc [Internet]. 2013;85:169–86. Available from: http://www.scielo.br/scielo.php?script=sci_arttext&pid=S0001-37652013000100169&lng=en&tlng=en

6. Barreira TF, Paula Filho GX, Rodrigues VCC, Andrade FMC, Santos RHS, Priore SE, et al. Diversidade e equitabilidade de Plantas Alimentícias Não Convencionais na zona rural de Viçosa, Minas Gerais, Brasil. Revista Brasileira de Plantas Medicinais [Internet]. 2015;17:964–74. Available from: http://www.scielo.br/scielo.php?script=sci_arttext&pid=S1516-05722015000600964&lng=pt&tlng=pt

7. Lima ILP, Scariot A, Medeiros MB de, Sevilha AC. Diversidade e uso de plantas do Cerrado em comunidade de Geraizeiros no norte do Estado de Minas Gerais, Brasil. Acta Bot Brasilica [Internet]. 2012;26:675–84. Available from: http://www.scielo.br/scielo.php?script=sci_arttext&pid=S0102-33062012000300017&lng=pt&tlng=pt

8. Brito MR de, Senna-Valle L de. Diversity of plant knowledge in a “Caiçara” community from the Brazilian Atlantic Forest coast. Acta Bot Brasilica [Internet]. 2012;26:735–47. Available from: http://www.scielo.br/scielo.php?script=sci_arttext&pid=S0102-33062012000400003&lng=en&tlng=en

9. Araújo FR, Lopes MA. Diversity of use and local knowledge of palms (Arecaceae) in eastern Amazonia. Biodivers Conserv [Internet]. 2012;21:487–501. Available from: http://link.springer.com/10.1007/s10531-011-0195-9

10. Campos LZ de O, Albuquerque UP, Peroni N, Araújo EL. Do socioeconomic characteristics explain the knowledge and use of native food plants in semiarid environments in Northeastern Brazil? J Arid Environ [Internet]. 2015;115:53–61. Available from: https://linkinghub.elsevier.com/retrieve/pii/S0140196315000038

11. Santos EA dos, Andrade L de HC. Conhecimento etnobotânico de moradores do Sítio Histórico de Olinda, Patrimônio Natural e Cultural da Humanidade. Rodriguésia [Internet]. 2020;71. Available from: http://www.scielo.br/scielo.php?script=sci_arttext&pid=S2175-78602020000100300&tlng=pt

12. Lobo RA de AM, Lobo ACBNM, de Oliveira AFM, Andrade L de HC. Ethnobotany as a parameter for the study of cultural mimicry among Roma people. Bol Latinoam Caribe Plantas Med Aromat [Internet]. 2022;21:530–47. Available from: https://blacpma.ms-editions.cl/index.php/blacpma/article/view/301/309

13. Silva AJ da R, Andrade L de HC. Etnobotânica nordestina: estudo comparativo da relação entre comunidades e vegetação na Zona do Litoral - Mata do Estado de Pernambuco, Brasil. Acta Bot Brasilica [Internet]. 2005;19:45–60. Available from: http://www.scielo.br/scielo.php?script=sci_arttext&pid=S0102-33062005000100006&lng=pt&tlng=pt

14. Silva HCH, Caraciolo RLF, Marangon LC, Ramos MA, Santos LL, Albuquerque UP. Evaluating different methods used in ethnobotanical and ecological studies to record plant biodiversity. J Ethnobiol Ethnomed [Internet]. 2014;10:48. Available from: https://ethnobiomed.biomedcentral.com/articles/10.1186/1746-4269-10-48

15. Mazon S, Menin D, Cella BM, Lise CC, Vargas T de O, Daltoé MLM. Exploring consumers’ knowledge and perceptions of unconventional food plants: case study of addition of Pereskia aculeata Miller to ice cream. Food Science and Technology [Internet]. 2020;40:215–21. Available from: http://www.scielo.br/scielo.php?script=sci_arttext&pid=S0101-20612020000100215&tlng=en

16. Souza RG DE, Dan ML, Dias-Guimarães MA, Guimarães LAOP, Braga JMA. Fruits of the Brazilian Atlantic Forest: allying biodiversity conservation and food security. An Acad Bras Cienc [Internet]. 2018;90:3583–95. Available from: http://www.scielo.br/scielo.php?script=sci_arttext&pid=S0001-37652018000703583&tlng=en

17. Bortolotto IM, Amorozo MC de M, Neto GG, Oldeland J, Damasceno-Junior GA. Knowledge and use of wild edible plants in rural communities along Paraguay River, Pantanal, Brazil. J Ethnobiol Ethnomed [Internet]. 2015;11:46. Available from: https://ethnobiomed.biomedcentral.com/articles/10.1186/s13002-015-0026-2

18. Nascimento VT do, de Lucena RFP, Maciel MIS, Albuquerque UP de. Knowledge and Use of Wild Food Plants in Areas of Dry Seasonal Forests in Brazil. Ecol Food Nutr [Internet]. 2013;52:317–43. Available from: http://www.tandfonline.com/doi/abs/10.1080/03670244.2012.707434

19. Cruz MP, Peroni N, Albuquerque UP. Knowledge, use and management of native wild edible plants from a seasonal dry forest (NE, Brazil). J Ethnobiol Ethnomed [Internet]. 2013;9:79. Available from: https://ethnobiomed.biomedcentral.com/articles/10.1186/1746-4269-9-79

20. Leal ML, Alves RP, Hanazaki N. Knowledge, use, and disuse of unconventional food plants. J Ethnobiol Ethnomed [Internet]. 2018;14:6. Available from: https://ethnobiomed.biomedcentral.com/articles/10.1186/s13002-018-0209-8

21. Nunes EN, Guerra NM, Arévalo-Marín E, Alves CAB, Nascimento VT do, Cruz DD da, et al. Local botanical knowledge of native food plants in the semiarid region of Brazil. J Ethnobiol Ethnomed [Internet]. 2018;14:49. Available from: https://ethnobiomed.biomedcentral.com/articles/10.1186/s13002-018-0249-0

22. Gomes DL, Ferreira RP dos S, Santos ÉM da C, Silva RRV da, Medeiros PM. Local criteria for the selection of wild food plants for consumption and sale: A case study in rural settlements in Alagoas, Brazil. Ethnobiology and Conservation [Internet]. 2020;9:1–15. Available from: https://ethnobioconservation.com/index.php/ebc/article/view/380/251

23. Conde BE, Ticktin T, Fonseca AS, Macedo AL, Orsi TO, Chedier LM, et al. Local ecological knowledge and its relationship with biodiversity conservation among two Quilombola groups living in the Atlantic Rainforest, Brazil. Melcher U, editor. PLoS One [Internet]. 2017;12:e0187599. Available from: https://dx.plos.org/10.1371/journal.pone.0187599

24. Medeiros PM de, Santos GMC dos, Barbosa DM, Gomes LCA, Santos ÉM da C, Silva RRV da. Local knowledge as a tool for prospecting wild food plants: experiences in northeastern Brazil. Sci Rep [Internet]. 2021;11:594. Available from: http://www.nature.com/articles/s41598-020-79835-5

25. Rodrigues E, Cassas F, Conde BE, da Cruz C, Barretto EHP, dos Santos G, et al. Participatory ethnobotany and conservation: a methodological case study conducted with quilombola communities in Brazil’s Atlantic Forest. J Ethnobiol Ethnomed [Internet]. 2020;16:2. Available from: https://ethnobiomed.biomedcentral.com/articles/10.1186/s13002-019-0352-x

26. Carneiro DB, Barboza MSL, Menezes MP. Plantas nativas úteis na Vila dos Pescadores da Reserva Extrativista Marinha Caeté-Taperaçu, Pará, Brasil. Acta Bot Brasilica [Internet]. 2010;24:1027–33. Available from: http://www.scielo.br/scielo.php?script=sci_arttext&pid=S0102-33062010000400017&lng=pt&tlng=pt

27. Pedrosa KM, de Almeida HA, Ramos MB, de Faria Lopes S. Plants with similar characteristics drive their use by local populations in the semi-arid region of Brazil. Environ Dev Sustain [Internet]. 2021;23:16834–47. Available from: https://link.springer.com/10.1007/s10668-021-01355-7

28. Chaves EMF, Silva JN, Lima A, Albuquerque UP, Barros RFM. Potential of wild food plants from the semi-arid region of northeast Brasil: chemical approach ethnoguided. Revista ESPACIOS [Internet]. 2015;36:1–9. Available from: www.revistaespacios.com/a15v36n16/15361620.html

29. Lucena RFP de, Medeiros PM de, Araújo E de L, Alves AGC, Albuquerque UP de. The ecological apparency hypothesis and the importance of useful plants in rural communities from Northeastern Brazil: An assessment based on use value. J Environ Manage [Internet]. 2012;96:106–15. Available from: https://linkinghub.elsevier.com/retrieve/pii/S0301479711003306

30. Luijk N Van, Soldati GT, Fonseca-Kruel VS da. The role of schools as an opportunity for transmission of local knowledge about useful Restinga plants: experiences in southeastern Brazil. J Ethnobiol Ethnomed [Internet]. 2021;17:34. Available from: https://ethnobiomed.biomedcentral.com/articles/10.1186/s13002-021-00461-0

31. Tuler AC, Peixoto AL, Silva NCB da. Plantas alimentícias não convencionais (PANC) na comunidade rural de São José da Figueira, Durandé, Minas Gerais, Brasil. Rodriguésia [Internet]. 2019;70. Available from: http://www.scielo.br/scielo.php?script=sci_arttext&pid=S2175-78602019000100271&tlng=pt

32. Cunha MA da, Paraguassú LAA, Assis JG de A, Silva AB de PC, Cardoso R de CV. Urban gardening and neglected and underutilized species in Salvador, Bahia, Brazil. J Ethnobiol Ethnomed [Internet]. 2020;16:67. Available from: https://ethnobiomed.biomedcentral.com/articles/10.1186/s13002-020-00421-0

33. Lucena CM de, Lucena RFP de, Costa GM, Carvalho TKN, Costa GG da S, Alves RR da N, et al. Use and knowledge of Cactaceae in Northeastern Brazil. J Ethnobiol Ethnomed [Internet]. 2013;9:62. Available from: https://ethnobiomed.biomedcentral.com/articles/10.1186/1746-4269-9-62

34. Alarcón JGS, Peixoto AL. Use of Terra Firme Forest by Caicubi Caboclos, Middle Rio Negro, Amazonas, Brazil. A Quantitative Study. Econ Bot [Internet]. 2008;62:60–73. Available from: http://link.springer.com/10.1007/s12231-007-9001-8

35. Lucena RFP, Albuquerque UP, Monteiro JM, Almeida CDFCBR, Florentino ATN, Ferraz JSF. Useful Plants of the Semi-Arid Northeastern Region of Brazil – A Look at their Conservation and Sustainable Use. Environ Monit Assess [Internet]. 2007;125:281–90. Available from: http://link.springer.com/10.1007/s10661-006-9521-1

36. Trotta J, Messias PA, Pires AHC, Hayashida CT, Camargo C de, Futemma C. Análise do conhecimento e uso popular de plantas de quintais urbanos no estado de São Paulo, Brasil. Revista de Estudos Ambientais. 2012;14:17–34.

37. Santos A da S dos, Oliveira LCL de, Curado FF, Amorim LO do. Caracterização e desenvolvimento de quintais produtivos agroecológicos na comunidade Mem de Sá, Itaporanga d’Ajuda-Sergipe Characterization and development of productive agroecological gardens in community Mem de Sá, Itaporanga d’Ajuda-Sergipe. Rev Bras de Agroecologia [Internet]. 2013 [cited 2022 Nov 6];8:100–11. Available from: https://www.bdpa.cnptia.embrapa.br/consulta/busca?b=ad&id=969200&biblioteca=vazio&busca=969200&qFacets=969200&sort=&paginacao=t&paginaAtual=1

38. Soldati GT, Duque-Brasil R, Da Silva TC, Coelho FMG, De Albuquerque UP. Conhecimento botânico e representações ambientais em uma comunidade rural no Domínio Atlântico: bases para conservação local. SITIENTIBUS série Ciências Biológicas [Internet]. 2012;11:265–78. Available from: http://periodicos.uefs.br/index.php/sitientibusBiologia/article/view/74

39. Silva N da, Farias R, Lucena P de, Lima JRDF, Deise G, Lima S, et al. Conhecimento e Uso da Vegetação Nativa da Caatinga em uma Comunidade Rural da Paraíba, Nordeste do Brasil. Bol Mus Biol Mello leitão (N sér). 2014;34:5–37.

40. Miranda TM, Hanazaki N. Conhecimento e uso de recursos vegetais de restinga por comunidades das ilhas do Cardoso (SP) e de Santa Catarina (SC), Brasil. Acta Bot Brasilica [Internet]. 2008;22:203–15. Available from: http://www.scielo.br/scielo.php?script=sci_arttext&pid=S0102-33062008000100020&lng=pt&tlng=pt

41. Rufino MU de L, Costa JT de M, Silva VA da, Andrade L de HC. Conhecimento e uso do ouricuri (Syagrus coronata) e do babaçu (Orbignya phalerata) em Buíque, PE, Brasil. Acta Bot Brasilica [Internet]. 2008;22:1141–9. Available from: http://www.scielo.br/scielo.php?script=sci_arttext&pid=S0102-33062008000400025&lng=pt&tlng=pt

42. Lucena CM de, Costa GM da, Sousa RF de, Carvalho TKN, Marreiros NDA, Alves CAB, et al. Conhecimento local sobre cactáceas em comunidades rurais na mesorregião do sertão da Paraíba (Nordeste, Brasil). Biotemas [Internet]. 2012;25. Available from: http://www.periodicos.ufsc.br/index.php/biotemas/article/view/20546

43. Strachulski J, Floriani N. Conhecimento popular sobre plantas: um estudo etnobotânico na comunidade rural de Linha Criciumal, em Cândido de Abreu-PR. Revista Geografar [Internet]. 2013;8:125–53. Available from: www.ser.ufpr.br/geografar

44. Florentino ATN, Araújo E de L, Albuquerque UP de. Contribuição de quintais agroflorestais na conservação de plantas da Caatinga, Município de Caruaru, PE, Brasil. Acta bot bras. 2007;21:37–47.

45. Gandolfo ES, Hanazaki N. Distribution of local plant knowledge in a recently urbanized area (Campeche District, Florianópolis, Brazil). Urban Ecosyst [Internet]. 2014;17:775–85. Available from: http://link.springer.com/10.1007/s11252-014-0345-4

46. Althaus-Ottmann MM, Cruz MJR da, Fonte NN da. Diversidade e uso das plantas cultivadas nos quintais do Bairro Fanny, Curitiba, PR, Brasil. Revista Brasileira de Biociências. 2011;9:39–49.

47. Hanazaki N, Tamashiro JY, Leitão-Filho HF, Begossi A. Diversity of plant uses in two Caiçara communities from the Atlantic Forest coast, Brazil. Biodivers Conserv. 2000;9:597–615.

48. Ribeiro JES, Carvalho TKN, Ribeiro JP de O, Guerra NM, Da Silva N, Pedrosa KM, et al. Ecological Apparency Hypothesis and Availability of Useful Plants: Testing different seu values. Ethnobotany Research & Applications [Internet]. 2014;12:415–32. Available from: www.ethnobotanyjournal.org/vol12/i1547-3465-12-415.pdf

49. Melo S, Lacerda VD, Hanazaki N. Espécies de restinga conhecidas pela comunidade do Pântano do Sul, Florianópolis, Santa Catarina, Brasil. Rodriguésia [Internet]. 2008;59:799–812. Available from: http://www.scielo.br/scielo.php?script=sci_arttext&pid=S2175-78602008000400799&lng=pt&tlng=pt

50. Alves CM, Lucena CM de, Santos S da S, Lucena RFP de, Trovão DM de BM. Ethnobotanical study of useful vegetal species in two rural communities in the semi-arid region of Paraíba state (Northeastern Brazil). Bol Mus Biol Mello Leitão. 2014;34:75–96.

51. Silva AAL, Tamashiro J, Begossi A. Ethnobotany of riverine populations from the Rio Negro, Amazonia (Brazil). J Ethnobiol. 2007;27:47–72.

52. Fernandes JM, Garcia FCP, Amorozo MC de M, Siqueira LC de, Marotta CPB, Cardoso IM. Etnobotânica de Leguminosae entre agricultores agroecológicos na Floresta Atlântica, Araponga, Minas Gerais, Brasil. Rodriguésia [Internet]. 2014;65:539–54. Available from: http://www.scielo.br/scielo.php?script=sci_arttext&pid=S2175-78602014000200015&lng=pt&tlng=pt

53. Gandolfo ES, Hanazaki N. Etnobotânica e urbanização: conhecimento e utilização de plantas de restinga pela comunidade nativa do distrito do Campeche (Florianópolis, SC). Acta Bot Brasilica [Internet]. 2011;25:168–77. Available from: http://www.scielo.br/scielo.php?script=sci_arttext&pid=S0102-33062011000100020&lng=pt&tlng=pt

54. Lopes LCM, Lobão AQ. Etnobotânica em uma comunidade de pescadores artesanais no litoral norte do Espírito Santo, Brasil. Bol Mus Biol Mello Leitão [Internet]. 2013 [cited 2022 Nov 6];29–52. Available from: https://redib.org/Record/oai_articulo638523-etnobot%C3%A2nica-em-uma-comunidade-de-pescadores-artesanais-litoral-norte-do-esp%C3%ADrito-santo-brasil

55. Fonseca-Kruel VS da, Peixoto AL. Etnobotânica na Reserva Extrativista Marinha de Arraial do Cabo, RJ, Brasil. Acta Bot Brasilica [Internet]. 2004;18:177–90. Available from: http://www.scielo.br/scielo.php?script=sci_arttext&pid=S0102-33062004000100015&lng=pt&tlng=pt

56. Miranda TM, Hanazaki N, Govone JS, Alves DMM. Existe utilização efetiva dos recursos vegetais conhecidos em comunidades caiçaras da Ilha do Cardoso, estado de São Paulo, Brasil? Rodriguésia [Internet]. 2011;62:153–69. Available from: http://www.scielo.br/scielo.php?script=sci_arttext&pid=S2175-78602011000100153&lng=pt&tlng=pt

57. Nascimento VT do, Vasconcelos MA da S, Maciel MIS, Albuquerque UP. Famine Foods of Brazil’s Seasonal Dry Forests: Ethnobotanical and Nutritional Aspects. Econ Bot [Internet]. 2012;66:22–34. Available from: http://link.springer.com/10.1007/s12231-012-9187-2

58. Poderoso RA, Hanazaki N, Junior AD. How is local knowledge about plants distributed among residents near a protected area? Ethnobiology and Conservation [Internet]. 2012;1. Available from: http://ethnobioconservation.com/index.php/ebc/article/view/15/90

59. Filho ESB, Santana MC de, Santos PAA, Ribeiro A de S. Levantamento etnobotânico da família Cactaceae no estado de Sergipe. Revista Fitos [Internet]. 2018;12. Available from: http://www.gnresearch.org/doi/10.5935/2446-4775.20180005

60. Zuchiwschi E, Fantini AC, Alves AC, Peroni N. Limitações ao uso de espécies florestais nativas pode contribuir com a erosão do conhecimento ecológico tradicional e local de agricultores familiares. Acta Bot Brasilica [Internet]. 2010;24:270–82. Available from: http://www.scielo.br/scielo.php?script=sci_arttext&pid=S0102-33062010000100029&lng=pt&tlng=pt

61. Lucena RFP de, Nascimento VT do, Lima Araújo E de, Albuquerque UP de. Local Uses of Native Plants in an Area of Caatinga Vegetation (Pernambuco, NE Brazil). Ethnobotany Research and Applications [Internet]. 2008;6:003. Available from: http://journals.sfu.ca/era/index.php/era/article/view/145

62. Pilla MAC, Amorozo MC de M. O conhecimento sobre os recursos vegetais alimentares em bairros rurais no Vale do Paraíba, SP, Brasil. Acta Bot Brasilica [Internet]. 2009;23:1190–201. Available from: http://www.scielo.br/scielo.php?script=sci_arttext&pid=S0102-33062009000400030&lng=pt&tlng=pt

63. Roque A de A, Loiola MIB. Potencial de uso dos recursos vegetais em uma comunidade rural no semiárido Potiguar. Revista Caatinga [Internet]. 2013 [cited 2022 Nov 6];26:88–98. Available from: https://periodicos.ufersa.edu.br/caatinga/article/view/2583

64. Cunha LVFC da, Albuquerque UP de. Quantitative Ethnobotany in an Atlantic Forest Fragment of Northeastern Brazil – Implications to Conservation. Environ Monit Assess [Internet]. 2006;114:1–25. Available from: http://link.springer.com/10.1007/s10661-006-1074-9

65. Eichemberg MT, Amorozo MC de M, Moura LC de. Species composition and plant use in old urban homegardens in Rio Claro, Southeast of Brazil. Acta Bot Brasilica [Internet]. 2009;23:1057–75. Available from: http://www.scielo.br/scielo.php?script=sci_arttext&pid=S0102-33062009000400016&lng=en&tlng=en

66. Albuquerque UP, Andrade LHC, Caballero J. Structure and floristics of homegardens in Northeastern Brazil. J Arid Environ [Internet]. 2005;62:491–506. Available from: https://linkinghub.elsevier.com/retrieve/pii/S0140196305000224

67. Santos LL dos, Nascimento ALB do, Vieira FJ, Silva VA da, Voeks R, Albuquerque UP. The Cultural Value of Invasive Species: A Case Study from Semi-Arid Northeastern Brazil. Econ Bot [Internet]. 2014 [cited 2022 Nov 6];1–18. Available from: https://www.jstor.org/stable/43305665

68. Baptista MM, Ramos MA, de Albuquerque UP, Coelho-de-Souza G, Ritter MR. Traditional botanical knowledge of artisanal fishers in southern Brazil. J Ethnobiol Ethnomed [Internet]. 2013;9:54. Available from: https://ethnobiomed.biomedcentral.com/articles/10.1186/1746-4269-9-54

69. Oliveira RLC de, Almeida LFP de, Scudeller VV, Barbosa RI. Traditional botanical knowledge variation between gender and age in a makuxi community in Roraima Savanna, northern Brazilian Amazonia. Ethnoscientia [Internet]. 2020;5. Available from: http://ethnoscientia.com/index.php/revista/article/view/340

70. Santos KL dos, Peroni N, Guries RP, Nodari RO. Traditional Knowledge and Management of Feijoa (Acca sellowiana) in Southern Brazil. Econ Bot [Internet]. 2009;63:204–14. Available from: http://link.springer.com/10.1007/s12231-009-9076-5

71. Neto EM de FL, Peroni N, Albuquerque UP de. Traditional Knowledge and Management of Umbu (Spondias tuberosa, Anacardiaceae): An Endemic Species from the Semi–Arid Region of Northeastern Brazil. Econ Bot [Internet]. 2010;64:11–21. Available from: http://link.springer.com/10.1007/s12231-009-9106-3

72. Arévalo-Marín E, Lima JR de F, Palma ART, Lucena RFP de, Cruz DD da. Traditional Knowledge in a Rural Community in the Semi-Arid Region of Brazil: Age and gender patterns and their implications for plant conservation. Ethnobotany Research and Applications [Internet]. 2015;14:331–44. Available from: http://journals.sfu.ca/era/index.php/era/article/view/1172

73. Pedrosa KM, Faria Lopes S de, Carvalho TKN, Lucena CM de, Lima­-Nascimento AM de, Lucena RFP de. Traditional management of Cactaceae by local populations in the semi­arid region of Brazil. Ethnobiology and Conservation [Internet]. 2020;2020:28. Available from: https://ethnobioconservation.com/index.php/ebc/article/view/349/268

74. Crepaldi MOS, Peixoto AL. Use and knowledge of plants by “Quilombolas” as subsidies for conservation efforts in an area of Atlantic Forest in Espírito Santo State, Brazil. Biodivers Conserv [Internet]. 2010;19:37–60. Available from: http://link.springer.com/10.1007/s10531-009-9700-9

75. Giraldi M, Hanazaki N. Use of Cultivated and Harvested Edible Plants by Caiçaras—What can Ethnobotany Add to Food Security Discussions? Human Ecology Review [Internet]. 2014;20. Available from: http://press-files.anu.edu.au/downloads/press/p291621/pdf/ch033.pdf

76. Milanesi L de S, Peroni N, dos Reis MS. Use of the palm Euterpe edulismartius in landscape units managed by migrants of German origin in Southern Brazil. J Ethnobiol Ethnomed [Internet]. 2013;9:47. Available from: https://ethnobiomed.biomedcentral.com/articles/10.1186/1746-4269-9-47

77. Christo AG, Guedes-Bruni RR, Fonseca-Kruel VS da. Uso de recursos vegetais em comunidades rurais limítrofes à Reserva Biológica de Poço das Antas, Silva Jardim, Rio de Janeiro: estudo de caso na Gleba Aldeia Velha. Rodriguésia [Internet]. 2006;57:519–42. Available from: http://www.scielo.br/scielo.php?script=sci_arttext&pid=S2175-78602006000300519&lng=pt&tlng=pt

78. Lucena CM de, Costa GGDS, Carvalho TKN, Guerra NM, Quirino ZGM, Lucena RFP de. Uso e conhecimento de cactáceas no município de São Mamede (Paraíba, nordeste do Brasil). Revista de Biologia e Farmácia. 2012;Especial:121–34.

79. Guerra NM, Ribeiro JE da S, Carvalho TKN, Pedrosa KM, Felix LP, Lucena RFP de. Usos locais de espécies vegetais nativas em uma comunidade rural no semiárido nordestino (São Mamede, Paraíba, Brasil). Revista de Biologia e Farmácia [Internet]. 2012;Especial:184–210. Available from: https://www.researchgate.net/publication/340660445
